# Adverse childhood experiences and functional stability in the affective-to-psychotic spectrum: a longitudinal multistate analysis of employment and relationship outcomes

**DOI:** 10.3389/fpsyt.2026.1930635

**Published:** 2026-10-06

**Authors:** Fanny Senner, Elena Merkle, Michael Lauseker, Eva C. Schulte, Urs Heilbronner, Monika Budde, Daniela Reich-Erkelenz, Mojtaba Oraki Kohshour, Alba Navarro-Flores, Maria Heilbronner, Ion-George Anghelescu, Volker Arolt, Bernhard T. Baune, Detlef E. Dietrich, Andreas J. Fallgatter, Christian Figge, Markus Jäger, Georg Juckel, Carsten Konrad, Nathalie Kühnen, Eva Z. Reininghaus, Max Schmauß, Andrea Schmitt, Simon Senner, Carsten Spitzer, Alkomiet Hasan, Peter Falkai, Thomas G. Schulze, Kristina Adorjan, Sergi Papiol, Sophie-Kathrin Greiner

**Affiliations:** 1 Institute of Psychiatric Phenomics and Genomics (IPPG), LMU University Hospital, LMU Medizin, LMU Munich, Munich, Germany; 2 Department of Psychiatry and Psychotherapy, LMU University Hospital, LMU Medizin, LMU Munich, Munich, Germany; 3 Centres for Psychiatry Suedwuerttemberg, Ravensburg, Germany; 4 Institute for Medical Information Processing, Biometry, and Epidemiology, Faculty of Medicine, LMU Munich, Munich, Germany; 5 Department of Psychiatry and Psychotherapy, Faculty of Medicine and University Hospital Bonn, University of Bonn, Bonn, Germany; 6 Institute of Human Genetics, University Hospital, Faculty of Medicine, University of Bonn, Bonn, Germany; 7 German Center for Mental Health (DZPG), Munich/Augburg, Germany; 8 Department of Immunology, Faculty of Medicine, Ahvaz Jundishapur University of Medical Sciences, Ahvaz, Iran; 9 International Max Planck Research School for Translational Psychiatry (IMPRS-TP), Munich, Germany; 10 Department of Psychiatry and Psychotherapy, Mental Health Institute Berlin, Berlin, Germany; 11 Institute for Translational Psychiatry, University of Münster, Münster, Germany; 12 Department of Psychiatry, University of Münster, Münster, Germany; 13 Department of Psychiatry, Melbourne Medical School, The University of Melbourne, Melbourne, VIC, Australia; 14 The Florey Institute of Neuroscience and Mental Health, The University of Melbourne, Parkville, VIC, Australia; 15 AMEOS Clinical Center Hildesheim, Hildesheim, Germany; 16 Center for Systems Neuroscience (ZSN), Hannover, Germany; 17 Department of Psychiatry, Medical School of Hannover, Hannover, Germany; 18 Department of Psychiatry and Psychotherapy, Tübingen Center for Mental Health (TüCMH), University of Tübingen, Tübingen, Germany; 19 German Center for Mental Health (DZPG), Partner Site Tübingen, Tübingen, Germany; 20 Karl-Jaspers Clinic, European Medical School Oldenburg-Groningen, Oldenburg, Germany; 21 Department of Psychiatry II, Ulm University, Bezirkskrankenhaus Günzburg, Günzburg, Germany; 22 Department of Psychiatry, Ruhr University Bochum, LWL University Hospital, Bochum, Germany; 23 Department of Psychiatry and Psychotherapy, Agaplesion Diakonieklinikum, Rotenburg, Germany; 24 Division of Psychiatry and Psychotherapeutic Medicine, Research Unit for Bipolar Affective Disorder, Medical University of Graz, Graz, Austria; 25 Clinic for Psychiatry, Psychotherapy and Psychosomatics, Augsburg University, Medical Faculty, Bezirkskrankenhaus Augsburg, Augsburg, Germany; 26 Laboratory of Neuroscience (LIM27), Institute of Psychiatry, University of Sao Paulo, São Paulo, Brazil; 27 Max Planck Institute of Psychiatry, Munich, Germany; 28 Center for Psychiatry Reichenau, Academic Hospital University of Konstanz, Konstanz, Germany; 29 Department of Psychosomatic Medicine and Psychotherapy, University Medical Center Rostock, Rostock, Germany; 30 Department of Psychiatry and Behavioral Sciences, SUNY Upstate Medical University, Syracuse, NY, United States; 31 Department of Psychiatry and Behavioral Sciences, Johns Hopkins University School of Medicine, Baltimore, MD, United States; 32 University Hospital of Psychiatry and Psychotherapy, University of Bern, Bern, Switzerland

**Keywords:** adverse childhood experience, employment, personal relationship, recovery, resilience, severe mental disorder

## Abstract

**Background:**

Adverse childhood experiences (ACEs) exert transdiagnostic influences on disorders of the affective-to-psychotic spectrum which share genetic similarities. While ACEs are established risk factors for developing disorders across this spectrum, the impact of ACEs on functional outcomes in adulthood remains insufficiently understood. This study aims to investigate the impact of ACEs on functional outcomes such as relationship and employment stability.

**Methods:**

We used multistate Markov models to analyze data from the longitudinal PsyCourse Study (N = 810) to investigate whether ACEs and ACE subtypes influence relationship and employment stability in individuals with affective-to-psychotic spectrum disorders and neurotypical controls. We analyzed whether protective factors (e.g., experience of feeling loved in childhood and polygenic resilience score for schizophrenia) modify these associations.

**Results:**

ACEs significantly increased the hazard of job loss (hazard ratio [HR] = 2.02, p = 0.0005), even after controlling for disease status and illness severity. Feeling loved in childhood emerged as a protective factor against job loss (HR = 0.52, p = 0.0096). For personal relationships, illness severity overshadowed ACE effects, with higher clinical severity rating predicting a higher hazard ratio for partnership separation. Among ACE subtypes, emotional abuse significantly predicted a higher hazard for partnership separation and lower for re-employment. Polygenic resilience score of schizophrenia did not contribute significantly.

**Discussion:**

In participants of the PsyCourse Study, ACEs substantially impact employment stability, personal relationship stability appears to be predominantly influenced by illness severity. These findings suggest the need for ACE-guided approaches in vocational rehabilitation and tailored interventions for partnership support in this population.

## 1 Introduction

Mental disorders along the affective-to-psychotic spectrum represent a significant subset of severe mental illnesses (SMI) and show substantial phenotypic and genetic overlap (1–3). Our understanding of these disorders has advanced considerably in recent decades, and research has shown that their etiology involves an interplay between genetic predisposition and psychosocial factors, particularly adverse childhood experiences (ACEs) (4, 5). Furthermore, treatment paradigms have shifted beyond symptom reduction toward a more holistic concept of recovery (6, 7). This perspective emphasizes patient-centered outcomes and acknowledges that affected individuals aspire to meaningful life goals, including stable personal relationships and securing sustainable employment (8).

According to the American Psychiatric Association Foundation, ACEs are “disruptions to the promotion of safe, stable, and nurturing family relationships and are characterized by stressful or traumatic events that occur during an individual’s first 18 years of life” (9). They occur across all social groups, educational levels, and ethnicities, though socioeconomically disadvantaged individuals are at higher risk (10, 11). Studies show that individuals with a history of ACEs have a threefold higher risk of developing psychotic and bipolar disorders (12, 13) and a more than two-fold risk for developing depression (14). In addition, when they develop a mental disorder, they tend to experience more severe symptoms, lower remission rates, and reduced cognitive performance (15–17).

Affective and psychotic disorders severely affect employment outcomes, and many patients lose their jobs after diagnosis (18). General employment rates in Europe range from 62% to 66%, but they are lower in people with bipolar disorders (40 -60%) and markedly lower in people with schizophrenia (10% to 20%) (19, 20). People with SMI show approximately a 10-fold reduction in job tenure compared to the general population (21). Employment is important, because it is associated with improved social functioning, self-esteem, symptom levels, and quality of life, and employed patients with SMI show higher rates of symptomatic remission and recovery (22).

Individuals with affective and psychotic disorders often struggle to maintain relationships: first-episode psychosis is linked to separation, bipolar disorder to higher divorce rates, and schizophrenia to difficulties forming new relationships (23–25). This situation is particularly concerning because life partners play a crucial role in recovery by supporting medication adherence, daily tasks, and early recognition of relapses (8, 26–28), which together ultimately improve prognosis (29).

In a previous PsyCourse Study paper, we found that patients with affective and psychotic disorders had a higher risk of job loss and relationship dissolution than neurotypical controls, partly mediated by diagnosis type and lower functioning (30). These findings prompted us to further examine the drivers of personal relationship and employment stability in the same PsyCourse study sample focusing on the aspect of ACEs.

Previous research found a compelling dose-response relationship between ACEs and impaired work performance and couple relationships in the general population. However, it remains unclear to what extent ACEs contribute to fluctuations in relationships of individuals with affective-to-psychotic spectrum disorders, and whether protective factors – both psychosocial and genetic – can buffer these effects. Benevolent childhood experiences, such as feeling loved and supported during childhood, have been conceptualized as a distinct dimension from the mere absence of adversity and have been shown to predict adult functional outcomes even in the presence of ACEs (31). In the present study, feeling loved during childhood was operationalized using a single reverse-coded item from the Childhood Trauma Screener that also contributes to the ACEs total score; while this precludes full independence between predictor and protective factor and does not capture the broader multidimensional construct of benevolent childhood experiences, it offers a pragmatic, transdiagnostically available indicator of perceived emotional support. Feeling loved during childhood has been proposed as a psychosocial resilience factor that may attenuate the impact of early adversity, while polygenic resilience scores (PRS) represent the first known approach to quantify genetic variant the promote resistance to schizophrenia (32). Given the substantial genetic overlap across psychotic-to-affective spectrum disorders, we investigated whether these two protective factors – feeling loved during childhood and SCZ-resilience PRS - reduce ACE-related fluctuations in employment and personal relationships, alongside examining the direct association between ACEs and these outcomes and potential subtype-specific effects. Beyond attachment-related pathways linking these vulnerabilities to relationship instability, ACEs may affect employment stability through distinct mechanisms, including impaired executive functioning and altered stress reactivity (33, 34), which can undermine workplace performance and interpersonal functioning at work.

Beyond cumulative ACE exposure, distinguishing between ACE subtypes may better capture their differential impact on adult functioning, as abuse (physical, emotional, sexual) and neglect (physical, emotional) have been linked to distinct developmental mechanisms—broadly, heightened threat sensitivity following abuse versus deficits in cognitive and reward-related processing following neglect (16, 35). We therefore examined the five ACE subtypes individually with respect to fluctuations in employment and relationship functioning.

To date, most studies on ACEs and adult functioning have relied on static measures of employment or relationship status, thereby providing limited insight into the dynamic processes underlying functional stability and instability over time. Multistate Markov models offer a powerful framework to model transitions between discrete functional states, such as employment, unemployment, partnership, and separation, and to estimate how ACEs influence the hazard of moving between these states (36).

Building on our previous findings, the present study aimed to investigate whether ACEs function as risk factors for fluctuations in professional and personal relationships and whether genetic and psychosocial protective factors buffer these vulnerabilities. Thus, we examined the following research questions:

1. Are ACEs associated with increased fluctuation in (a) employment and (b) personal relationships?
2. Do protective factors (feeling loved during childhood and polygenic resilience score for schizophrenia (SCZ-resilience PRS)) reduce fluctuations in (a) employment and (b) personal relationships?
3. Do different ACE subtypes show distinct associations with (a) employment stability and (b) personal relationship stability?

By examining how ACEs, protective factors, and relationship outcomes interact, this study sought to clarify mechanisms shaping social and occupational functioning in mental disorders and to inform targeted interventions that support stable relationships and employment.

## 2 Participants and methods

### 2.1 Study participants

For this study, we analyzed data (version 6.0) (37) from the PsyCourse Study, a longitudinal, naturalistic, multi-center research project performed in Germany and Austria from 2012 to 2019 (38). The PsyCourse Study aims to investigate clinical, neurobiological, and genetic factors that influence the long-term progression of SMIs. It encompassed an extensive range of phenotypic assessments and biological sample collection at up to four study visits equally spaced over 18 months (baseline, 6, 12, and 18 months). A comprehensive description of the study design is available in Budde et al. (39).

Psychiatric diagnoses were confirmed with parts of the Structured Clinical Interview for DSM-IV (SCID) (40), and neurotypical controls were evaluated with the Mini-DIPS (41). From the original 1,786 participants, 810 were included based on the following criteria: <65 years at baseline, ≥2 study visits, and completion of the Childhood Trauma Screener (CTS) (42) at visit 3. Genotyping data were available for all 810 included participants; thus, genotyping status did not further reduce the analytic sample. The sample included 571 participants with affective-to-psychotic spectrum disorders (70.5%) and 239 controls without psychiatric diagnoses (29.5%). All participants gave written informed consent, and the study was ethics-approved and conducted in accordance with the Helsinki Declaration (1975/2000).

### 2.2 Phenotypic data

Our study included phenotypic data (37) on sex, age, study site, study visit, DSM-IV diagnosis, disease status (clinical vs. control), educational level, and symptom severity as measured by the Clinical Global Impressions (CGI) scale (43). Educational level was assessed on a scale, which combines information on high-school and professional educational level, ranging from 0 to 6 (where 0 indicated the educational level and 6, the highest). Relationship and employment status were recorded and categorized within the German work and pension system, which may not fully align with other countries. To enhance clarity, the study therefore used “currently in a relationship” and “currently in paid employment”, and excluded participants who were only occasionally employed (n = 18). Additionally, ACEs were assessed with the CTS.

Participants were assessed up to four times at six-month intervals. At each study visit, all the above-mentioned phenotypic data were assessed, except for the CTS, which was assessed only at visit 3. The number of participants lost to follow-up increased over time, particularly approaching the fourth study visit (**Table 1**).

**Table 1:** Descriptive characteristics of the sample.

Variable	Total (n = 810)	Clinical (n =571)	Control (n = 239)	Statistic	p-value
Age, y	40.8 (SD, 12.8)	43.3 (SD, 11.5)	34.8 (SD, 13.7)	W = 93752	p < 0.001
Sex, female	412 (50.9%)	263 (46.0%)	149 (62.3%)	X² = 17.9	p < 0.001
ACE*					
Any type of ACE				X² = 43.9	p < 0.001
Y	344 (42.5%)	285 (49.9%)	59 (24.7%)		
N	466 (57.5%)	286 (50.0%)	180 (75.3%)		
NA	0 (0%)	0 (0%)	0 (0%)		
Emotional neglect				X² = 25.4	p < 0.001
Y	137 (16.9%)	121 (21.2%)	16 (6.7%)		
N	671 (82.8%)	448 (78.5%)	223 (93.3%)		
NA	2 (0.3%)	2 (0.4%)	0 (0%)		
Physical abuse				X² = 14.7	p < 0.001
Y	125 (15.4%)	106 (18.6%)	19 (8.0%)		
N	683 (84.3%)	463 (81.4%)	220 (92.1%)		
NA	2 (0.3%)	2 (0.4%)	0 (0%)		
Emotional abuse				X² = 21.6	p < 0.001
Y	175 (21.6%)	148 (26.0%)	27 (11.3%)		
N	632 (78.0%)	420 (73.6%)	212 (88.7%)		
NA	3 (0.4%)	3 (0.5%)	0 (0%)		
Sexual abuse				X² = 9.9	p = 0.002
Y	128 (15.8%)	105 (18.4%)	23 (9.6%)		
N	678 (83.7%)	462 (80.9%)	216 (90.4%)		
NA	4 (0.5%)	4 (0.7%)	0 (0%)		
Physical neglect				X² = 15.3	p < 0.001
Y	87 (10.7%)	77 (13.5%)	10 (4.2%)		
N	722 (89.1%)	493 (86.3%)	229 (95.8%)		
NA	1 (0.1%)	1 (0.2%)	0 (0%)		
Educational status				W = 44238	p < 0.001
0	6 (0.7%)	6 (1.0%)	0 (0%)		
1	29 (3.6%)	28 (4.9%)	1 (0.4%)		
2	108 (13.3%)	102 (17.9%)	6 (2.5%)		
3	217 (26.8%)	140 (24.5%)	77 (32.2%)		
4	125 (15.4%)	105 (18.4%)	20 (8.4%)		
5	86 (10.6%)	62 (10.9%)	24 (10.0%)		
6	220 (27.16%)	112 (19.6%)	108 (45.2%)		
NA	19 (2.35%)	16 (2.8%)	3 (1.3%)		
CGI					
1		6 (0.7%)	239 (29.5%)		
2		30 (3.7%)			
3		151 (18.6%)			
4		168 (20.7%)			
5		186 (23.0%)			
6		23 (2.8%)			
7		1 (0.1%)			
NA		6 (0.7%)			
CGI category**					
Healthy/mildly ill	275 (33.9%)				
Moderately ill	319 (39.3%)				
Severely ill	210 (25.9%)				

Variable	Total	Clinical	Control	Statistics	p-value
Employed?					
Baseline				X² = 71.4	p < 0.001
Y	419 (51.7%)	240 (42.0%)	179 (74.9%)		
N	387 (47.8%)	327 (57.3%)	60 (25.1%)		
NA	4 (0.5%)	4 (0.7%)	0 (0%)		
6 months				X² = 98.8	p < 0.001
Y	404 (49.9%)	225 (39.4%)	179 (74.9%)		
N	307 (37.9%)	274 (48.0%)	33 (13.8%)		
NA	99 (12.2%)	72 (12.6%)	27 (11.3%)		
12 months				X² = 119.9	p < 0.001
Y	431 (53.2%)	236 (41.3%)	195 (81.6%)		
N	364 (44.9%)	324 (56.7%)	40 (16.7%)		
NA	15 (1.9%)	11 (1.9%)	4 (1.7%)		
18 months				X² = 77.3	p < 0.001
Y	361 (44.6%)	192 (33.6%)	169 (70.7%)		
N	273 (33.7%)	234 (41.0%)	39 (16.3%)		
In a partnership?					
Baseline				X² = 28.9	p < 0.001
Y	409 (50.5%)	252 (44.1%)	157 (65.7%)		
N	391 (48.3%)	309 (54.1%)	82 (34.3%)		
NA	10 (1.2%)	10 (1.8%)	0 (0%)		
6 months				X² = 30.6	p < 0.001
Y	369 (45.6%)	222 (38.9%)	147 (61.5%)		
N	339 (41.9%)	269 (47.1%)	70 (29.3%)		
NA	102 (12.6%)	80 (14.0%)	22 (9.2%)		
12 months				X² = 27.1	p < 0.001
Y	406 (50.1%)	250 (43.8%)	156 (65.3%)		
N	383 (47.3%)	301 (52.7%)	82 (34.3%)		
NA	21 (5.6%)	20 (3.5%)	1 (0.4%)		
18 months				X² = 24.3	p < 0.001
Y	336 (41.5%)	194 (34.0%)	142 (59.4%)		
N	299 (36.9%)	228 (29.9%)	71 (29.7%)		
NA	175 (21.6%)	149 (26.1%)	26 (10.9%)		

All results are shown as n (%), except for age, which is shown as mean (SD).

*ACE measured by Childhood Trauma Screener with cut-off values established by Glaesmer et al. (44).

**Clinical Global Impressions scale categories: scale (CGI scores were grouped into three categories: (1) 1–2 = healthy/mildly ill; (2) 3–4 = moderately ill; (3) 5–7 = severely ill); ACE, adverse childhood experience; CGI, Clinical Global Impressions scale; N, no; NA, not available; SD, standard deviation; W, Mann-Whitney *U* value; X², chi-squared test; Y, yes. Significance level: p-value < 0.05.

CGI scale: The CGI is a widely used clinician-rated scale of illness severity ranging from 1 (“normal, not at all ill”) to 7 (“among the most extremely ill patients”) (43). Baseline CGI scores were used; controls were assigned a score of 1, and patient scores were grouped as 1–2 (“healthy/mildly ill”), 3–4 (“moderately ill”), and 5–7 (“severely ill”).

CTS: The CTS is a brief version of the Childhood Trauma Questionnaire (42), with each item corresponding to one of five ACE subtypes and rated on a 5-point Likert scale ranging from “never” to “very often”. It consists of a self-report, assessing ACE subtypes include emotional neglect, physical abuse, emotional abuse, sexual abuse, and physical neglect. The CTS has demonstrated good convergent validity with the corresponding CTQ subscales (r = .55–.87) and adequate internal consistency (Cronbach’s α = .76) (42). To determine the presence of ACEs, we applied the cut-off values established by Glaesmer et al. (≥3 for emotional and physical abuse, ≥2 for sexual abuse, and ≥4 for emotional and physical neglect) (45). To assess the experience of feeling loved (research question 2), the first item (emotional neglect) of the CTS was used in reverse-coded, dichotomous form.

### 2.3 Genetic analyses

Participants underwent genotyping with Illumina’s Global Screening Array version 3.0 (Life & Brain GmbH). Genetic analyses were performed as described in Greiner et al. (46), and genotype dosage data were used to compute polygenic resilience score for schizophrenia (SCZ-resilience PRS) by using summary statistics from corresponding genome-wide association studies (32). Note: Note: In the present work, PRS refers to the schizophrenia polygenic resilience score not to a conventional polygenic risk score. The SCZ-resilience PRS was developed to index genetic variation associated with relative resilience among individuals with elevated polygenic liability for schizophrenia and is therefore not simply the inverse of a schizophrenia risk score. Because psychiatric disorders show substantial genetic overlap, this score may also capture broader, transdiagnostic resilience-related mechanisms, although such cross-disorder interpretations remain exploratory. For more information, see **Supplementary Material 1**.

### 2.4 Statistical analysis

Descriptive data were analyzed with JASP for Windows, version 0.18.3. Descriptive statistics included means and standard deviations for continuous data and frequencies and percentages for categorical variables. Normality was assessed using the Shapiro-Wilk test and Q-Q plots. Group differences were analyzed with the two-sided independent t test for normally distributed data. Mann-Whitney U tests otherwise, and the chi-square test for categorical variables.

All analyses regarding the three research questions were performed with R (47). In addition to standard R packages, the msm package was used to fit multistate Markov models (36); following a procedure similar to that described by Senner et al. (30). The multi-state Markov model is a useful way of describing a process in which an individual moves through a series of states in continuous time. Group comparisons were conducted using two-stage multistate models in which both states were treated as transient, allowing participants to move repeatedly between them. This approach accommodated incomplete data, provided that participants had attended at least two study visits, because transitions were estimated from available observations. Two model series were specified: (1) employment status transitions, based on the binary variable “currently in paid employment,” and (2) relationship status transitions, based on the binary variable “currently in a relationship.” Both were specified within a Markovian framework, such that transition probabilities depended only on the current state. Risk differences were evaluated using hazard ratios (HRs), comparing participants with versus without the respective exposure, with HR > 1 indicating increased risk an HR < 1 indicating reduced risk of transition in the exposed group relative to the unexposed group. Time spent in each state was modeled using an exponential distribution. Study visit number served as the time scale, as this yielded only minor differences compared with models using exact timestamps. Analyses focused on transition intensities rather than state occupation probabilities.

Each series comprised a basic and extended multistate model which included predefined variable sets that were combined to evaluate their effects on HRs. The basic model included ACEs, defined as values above the cut-off in at least one ACE domain, or feeling loved in childhood; SCZ-resilience PRS; age, sex, and, where applicable, educational level; and ancestry principal components 1 and 2, which capture major axes of genetic variation related to population ancestry and were included to control for potential population stratification in the PRS analyses. The extended model included all basic model variables plus clinical vs. control status and CGI (illness severity). Research questions 1 and 2 were examined in the full sample of this study (n=810), whereas research question 3 was addressed only among individuals with a history of ACE (n = 344).

Statistical significance was set at α = 0.05. No multiple-testing correction was applied because pre-specified models were tested for each outcome with adjustment for confounders (48).

## 3 Results

### 3.1 Descriptive analyses

At the first interview, mean age was 40.8 years (SD 12.8), and the sex distribution was nearly equal (50.9% women, 49.1% men). Overall, 42.5% of participants reported any ACE with higher rates in patients (49.9%) than in neurotypical controls (24.7%). The clinical group included individuals diagnosed with schizophrenia (n = 223; 39.1%), schizoaffective disorder (n = 47; 8.2%), schizophreniform disorder (n = 4; 0.7%), brief psychotic disorder (n = 2; 0.4%), bipolar I disorder (n = 218; 38.2%), bipolar II disorder (n = 43; 7.5%), and recurrent unipolar depression (n = 34; 6.0%). **Table 1** provides further descriptive data, including employment and relationship status.

### 3.2 Multistate models

#### 3.2.1 Research question 1 (risk factor “ACEs”)

##### 3.2.1.1 Employment transitions

**Figure 1:**
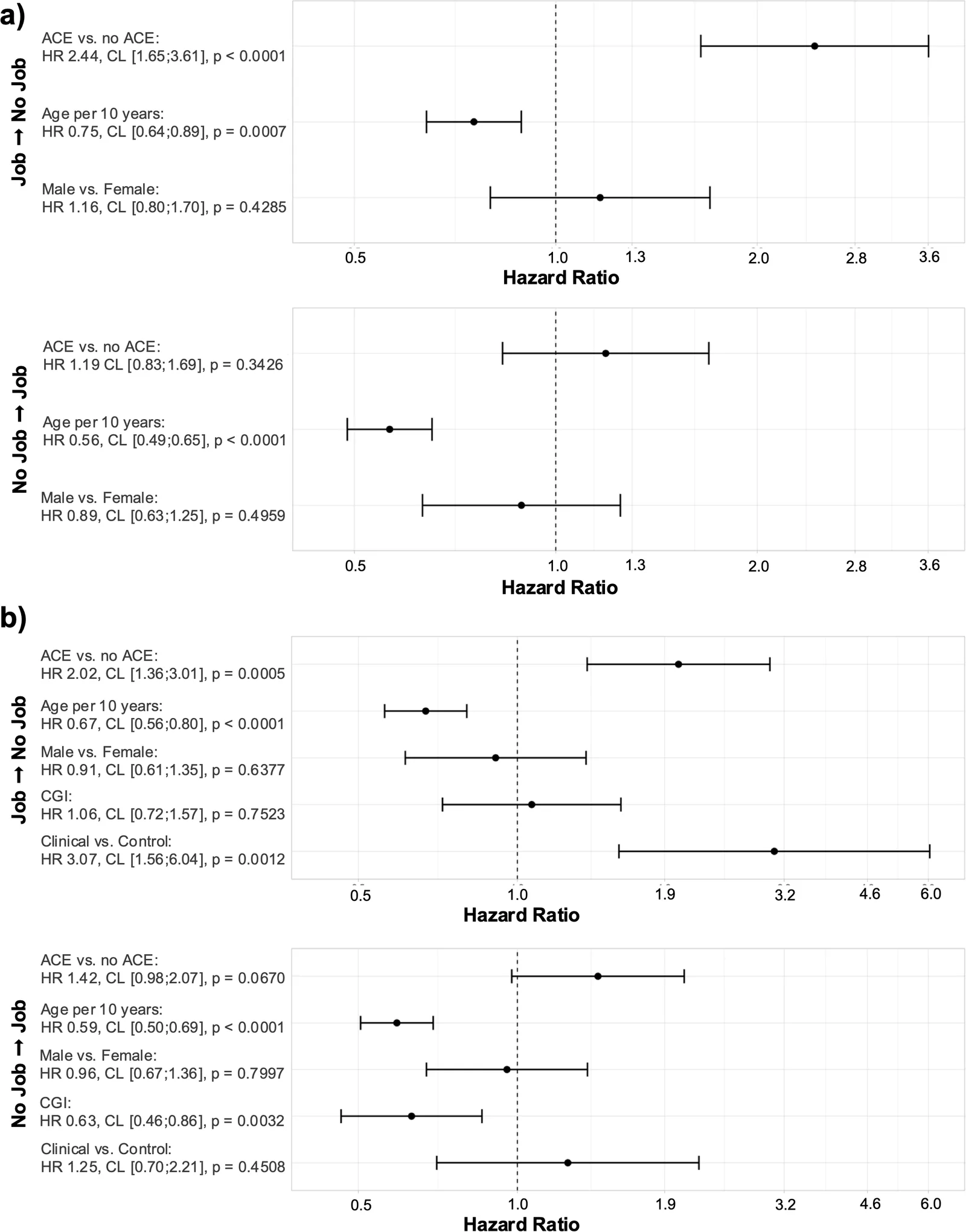
Hazard ratios of losing or finding a job in the group exposed to adverse childhood experiences and in the reference group. **(a)** Basic multistate models (corrected for age and sex); **(b)** extended multistate models (corrected for age, sex, Clinical Global Impressions score, and clinical vs. control status). Significance level: p-value < 0.05. ACE, adverse childhood experience; CGI, Clinical Global Impressions scale (CGI scores were grouped into three categories: (1) 1–2 = healthy/mildly ill; (2) 3–4 = moderately ill; (3) 5–7 = severely ill); CI, confidence interval; HR, hazard ratio.

In the basic multistate model (**Figure 1**), ACE exposure was associated with a higher hazard of job loss (hazard ratio, HR = 2.44; 95% CI [1.65; 3.61]; p < 0.0001) but not with re-employment.

In the extended model (**Figure 1**), this association remained (HR = 2.02; 95% CI [1.36; 3.01]; p = 0.0005), and the clinical group showed a higher hazard of job loss than controls (HR = 3.07; 95% CI [1.56; 6.04]; p = 0.0012). Re-employment was unrelated to ACE exposure but decreased with higher CGI scores (HR = 0.63; 95% CI [0.46; 0.86]; p = 0.0032). ACEs was again not associated with re-employment, whereas higher CGI scores showed a lower hazard of re-employment (HR = 0.63; 95% CI [0.46; 0.86]; p = 0.0032).

No other covariates were significant. Across models, older age was associated with lower hazards of both job loss and re-employment; educational level was not significant where included (**Supplementary Material Figure 1**).

##### 3.2.1.2 Partnership transitions

**Figure 2:**
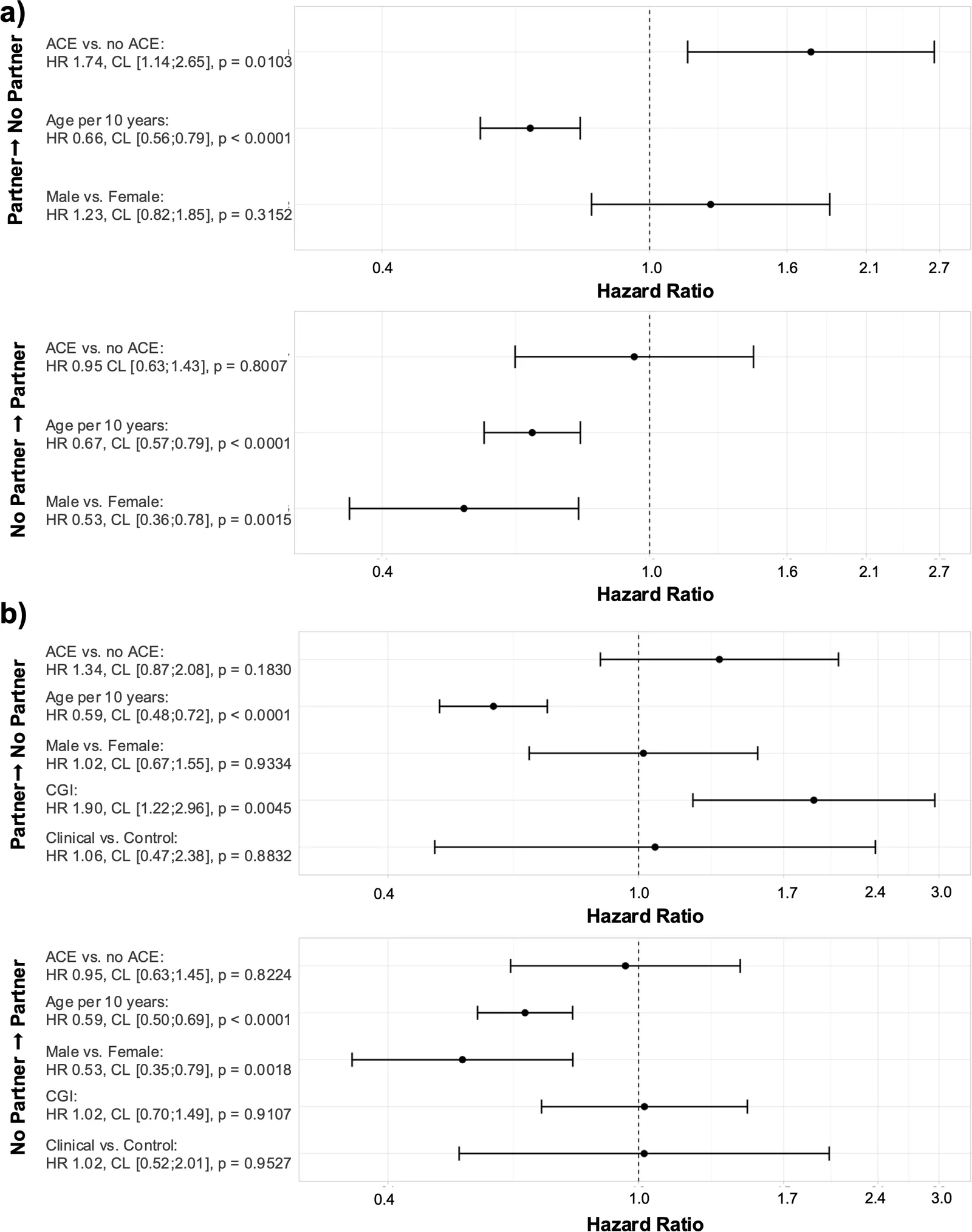
Hazard ratios of losing or finding a partner in the group exposed to adverse childhood experiences and in the reference group. **(a)** Basic multistate models (corrected for age and sex); **(b)** extended multistate models (corrected for age, sex, Clinical Global Impressions score, and clinical vs. control status). Significance level: p-value < 0.05. ACE, adverse childhood experience; CGI, Clinical Global Impressions scale (CGI scores were grouped into three categories: (1) 1–2 = healthy/mildly ill; (2) 3–4 = moderately ill; (3) 5–7 = severely ill); CI, confidence interval; HR, hazard ratio.

In the basic models (**Figure 2**), ACE exposure was associated with a higher hazard of separation (HR = 1.74; 95% CI [1.14; 2.65]; p = 0.0103) but not with forming a new partnership. Male sex predicted a lower hazard of forming a new partnership (HR = 0.53; 95% CI [0.36; 0.78]; p = 0.0015).

In the extended models (**Figure 2**), higher CGI scores were associated with a higher hazard of separation (HR = 1.90; 95% CI [1.22; 2.96]; p = 0.0045). Men again had a lower hazard of forming a new partnership (HR = 0.53; 95% CI [0.35; 0.79]; p = 0.0018).

No other covariates were significant. Across models, older age was associated with lower hazards of both separation and forming new partnerships.

#### 3.2.2 Research question 2 (protective factors “feeling loved in childhood” and “SCZ-resilience PRS”)

##### 3.2.2.1 Employment transitions

**Figure 3:**
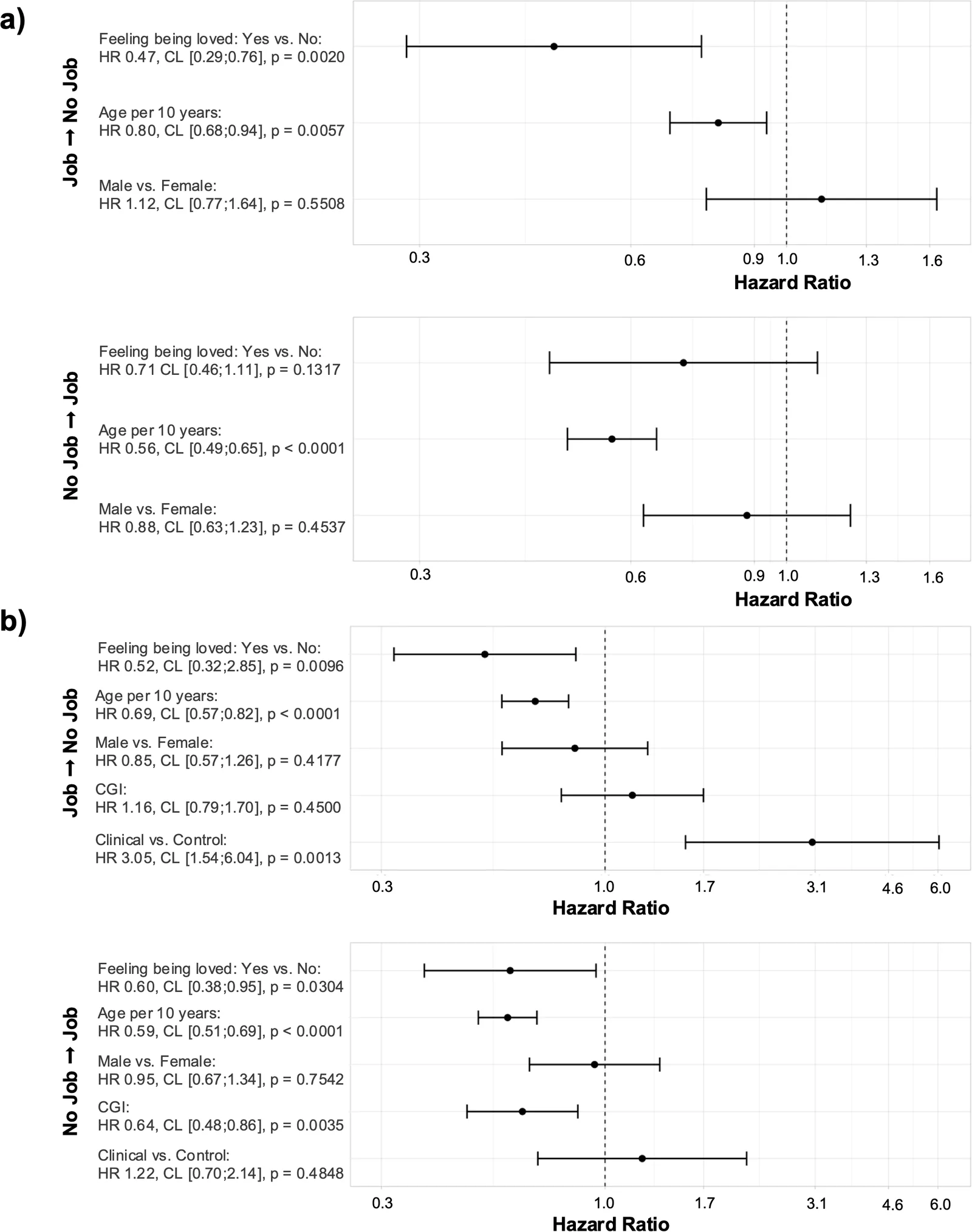
Hazard ratios of losing or finding a job in the group exposed to adverse childhood experiences and in the reference group. **(a)** Basic multistate models (corrected for age and sex); **(b)** extended multistate models (corrected for age, sex, Clinical Global Impressions score, and clinical vs. control status). Significance level: p-value < 0.05. CGI, Clinical Global Impressions scale (CGI scores were grouped into three categories: (1) 1–2 = healthy/mildly ill; (2) 3–4 = moderately ill; (3) 5–7 = severely ill); CI, confidence interval; HR, hazard ratio.

In the basic models (**Figure 3**), feeling loved in childhood was associated with a lower hazard of job loss (HR = 0.47; 95% CI [0.29; 0.76]; p = 0.0020) but not with re-employment.

In the extended models (**Figure 3**), feeling loved remained protective for job loss (HR = 0.52; 95% CI [0.32; 0.85]; p = 0.0096), and the clinical group had a higher hazard of job loss than controls (HR = 3.05; 95% CI [1.54; 6.04]; p = 0.0013). For re-employment, feeling loved (HR = 0.60; 95% CI [0.38; 0.95]; p = 0.0304) and higher CGI scores (HR = 0.64; 95% CI [0.48; 0.86]; p = 0.0035) were associated with a lower hazard of re-employment.

No other covariates were significant. Across all models, older age predicted lower hazards of both job loss and re-employment, and educational level and SCZ-resilience PRS were not significant where included (**Supplementary Material Figures 2, 3**).

##### 3.2.2.2 Partnership transitions

**Figure 4:**
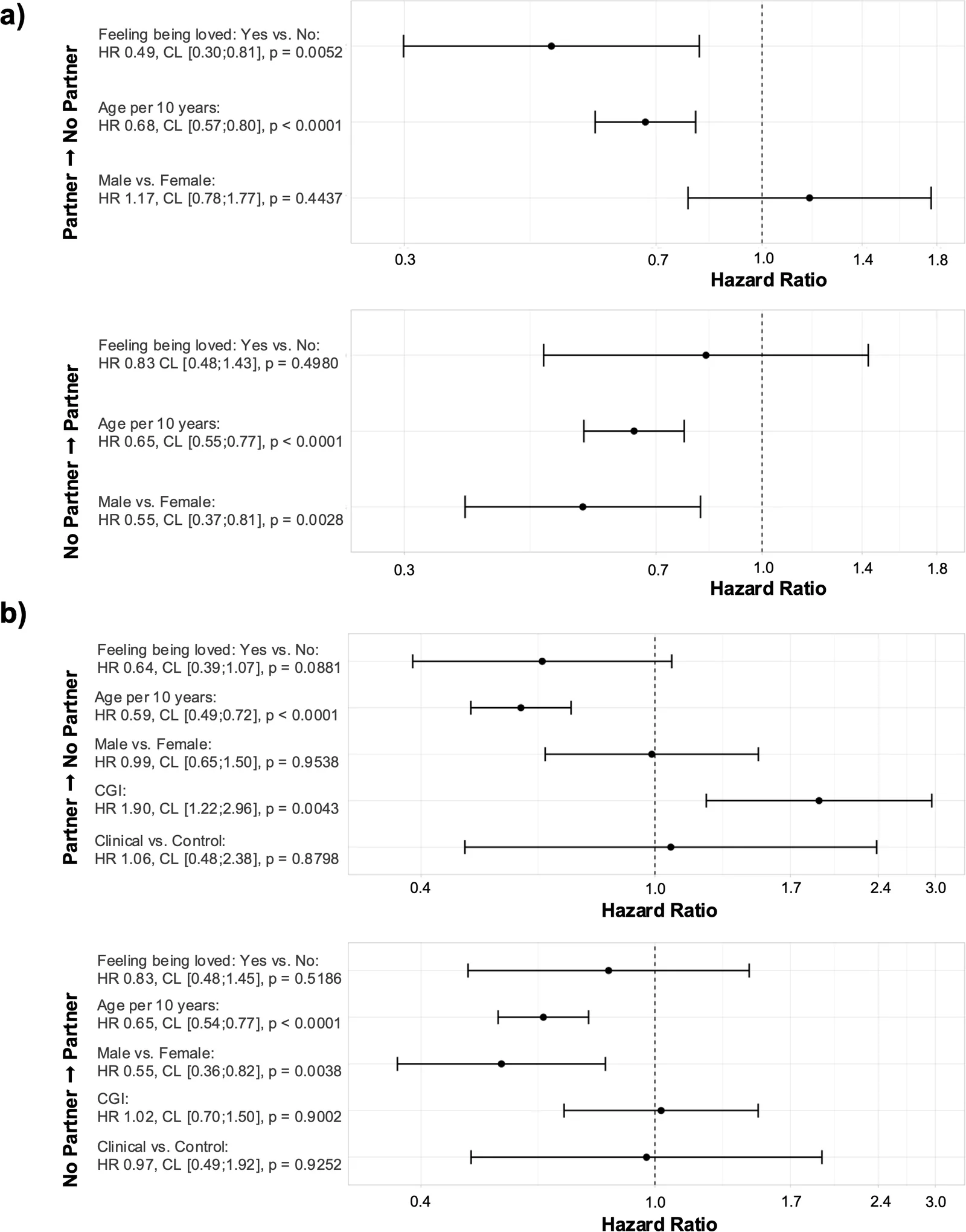
Hazard ratios of losing or finding a partner in the group exposed to adverse childhood experiences and in the reference group. **(a)** Basic multistate models (corrected for age and sex); **(b)** extended multistate models (corrected for age, sex, Clinical Global Impressions score, and clinical vs. control status). Significance level: p-value < 0.05. CGI, Clinical Global Impressions scale (CGI scores were grouped into three categories: (1) 1–2 = healthy/mildly ill; (2) 3–4 = moderately ill; (3) 5–7 = severely ill); CI, confidence interval; HR, hazard ratio.

In the basic models (**Figure 4**), feeling loved in childhood was associated with a lower hazard of separation (HR = 0.49; 95% CI [0.30; 0.81]; p = 0.0042) but not with finding a new partner. Men had a lower hazard of finding a new partner than women (HR = 0.55; 95% CI [0.37; 0.81]; p = 0.0028).

In the extended models (**Figure 4**), feeling loved was no longer significant for separation; instead, higher CGI scores predicted a higher hazard of separation (HR = 1.90; 95% CI [1.22; 2.96]; p = 0.0043). Men again had a lower hazard of finding a new partner (HR = 0.55; 95% CI [0.36; 0.82]; p = 0.0038).

No other covariates were significant. Across all models, older age was associated with lower hazards of both separation and forming new partnerships, and SCZ-resilience PRS was not significant when included (**Supplementary Material Figure 4**).

#### 3.2.3 Research question 3 (risk factor “ACE subtypes” among individuals with ACEs)

This subanalysis was restricted to participants with a history of ACEs (n = 344/810).

##### 3.2.3.1 Employment transitions

Only emotional abuse was associated with a lower hazard of re-employment (HR = 0.56; 95% CI [0.33; 0.95]; p = 0.0305) in both the basic and extended models. No other ACE subtype was associated with job loss or re-employment (**Supplementary Material: Table 1**; **Figure 5**).

##### 3.2.3.2 Partnership transitions

Only emotional abuse was associated with a higher hazard of separation in both the basic (HR = 2.23; 95% CI [1.23; 4.41]; p = 0.0096) and extended models (HR = 2.06; 95% CI [1.08; 3.94]; p = 0.0285). No other ACE subtype showed significant associations (**Supplementary Material: Table 2**; **Figure 6**).

## 4 Discussion

In our study, we analyzed the influence of ACEs and protective factors on fluctuations between different states of personal relationships and employment over time. We found that ACEs particularly increased the hazard of job loss, feeling loved in childhood promoted employment stability, and that illness severity and emotional abuse were associated with partnership dissolution. Yet, the protective SCZ-resilience PRS did not influence relationship fluctuations.

In the analysis of occupational relationships in patients and neurotypical controls, ACEs emerged as a risk factor for job loss and feeling loved in childhood emerged as a protective factor for occupational stability. These findings align with the attachment theory (49): safe, stable, and nurturing relationships have been linked to better health outcomes, reduced mental distress (50, 51) and greater satisfaction in adult romantic relationships (52), whereas early abuse and neglect are linked to lower educational attainment and wages and higher unemployment in midlife (53, 54). While the link between ACEs and relationship instability is well explained by attachment theory, the pathways linking ACEs to employment instability are less direct but similarly plausible. ACEs have been associated with impaired executive functioning, including working memory and inhibitory control, and with dysregulation of the hypothalamic-pituitary-adrenal (HPA) axis and altered stress reactivity in adulthood (33, 34), both of which are critical for sustained task performance, reliability, and adaptability in the workplace. Additionally, emotion dysregulation following early adversity —already implicated in interpersonal difficulties in romantic relationships— may similarly affect relationships with colleagues and supervisors, increasing the risk of conflict or job loss. Interestingly, feeling loved in childhood was also linked to a lower hazard of re-employment. possibly reflecting greater job stability, as these individuals may retain jobs longer and thus less often need to seek new employment.

ACEs showed a stronger and partly distinct impact on occupational than on personal relationships. At work, ACE effects remained after controlling for illness severity, indicating a direct, independent influence on workplace stability. In partnerships, ACEs predicted separation in basic models, but not after adjusting for illness severity in extended models, suggesting that illness severity is the more proximal driver of intimate relationship stability and that ACEs act indirectly via increased severity (15).

SMI place a particular burden on partnerships (55, 56). Caregivers distress may create dyadic strain that contributes to separation (57). Unlike professional settings, personal relationships remain continuously exposed to illness-related stressors, which may help explain why illness severity is closely linked to partnership instability (58, 59).

To identify the most influential ACE subtype, we conducted a subanalysis in participants reporting ACEs (n = 344/810). In this trauma-exposed subgroup, emotional abuse predicted separation and lower re-employment hazards more strongly than other ACE subtypes. As subtype-specific hazard ratios were estimated in separate Markov models rather than compared directly through formal statistical tests, this finding should be interpreted with caution and requires replication in larger samples. Nonetheless, it is consistent with evidence linking emotional abuse to impaired adult romantic functioning (60) and emotion dysregulation (61), which can hinder emotional processing and contribute to interpersonal difficulties through maladaptive, response-focused regulation strategies (62). These mechanisms may partly explain the observed instability in both private and occupational relationships. These mechanisms may partly explain the observed instability in both private and occupational relationships.

Regarding the demographic variables, and in line with our previous study (30) older age had an overall stabilizing effect on all relationships. Younger individuals and women had higher chances of finding a new partner, whereas SCZ-resilience PRS showed no significant effect. Educational status also did not appear to influence professional relationship stability, although other research identified educational attainment as a mediator between ACEs and unemployment (53).

The influence of SCZ-resilience PRS on relationship and employment fluctuations was not significant, in contrast to the protective effect of feeling loved during childhood. This may partly reflect limited statistical power, as PRS effects are typically small and null findings often stem from insufficient sample size rather than a true absence of effect (63). Additionally, the SCZ-specific PRS may not fully capture resilience mechanisms relevant across the broader affective-to-psychotic spectrum, and as a distal genetic factor, its behavioral expression may be more easily overshadowed by proximal factors such as illness severity in a clinical sample with manifest psychopathology. Educational status showed a similarly non-significant effect, possibly reflecting its more distal relationship to day-to-day functional fluctuations.

Strengths include the large sample, longitudinal and transdiagnostic design (affective-to-psychotic spectrum plus neurotypical controls), and multistate models capturing relationship fluctuations rather than static states, alongside integration of phenotypic and genetic data. Limitations include missing reasons for relationship changes (and whether dissolutions were beneficial), potential bias from substantial 12- and 18-month dropout (especially among outpatients). Relatedly, participants excluded due to insufficient follow-up visits or incomplete CTS assessment at visit 3 could not be systematically compared with the analytic sample regarding baseline demographic, clinical, and functional characteristics, limiting our ability to formally assess selection bias; however, the exclusion criteria (fewer than two visits, missing CTS data) likely reflect general study attrition rather than systematic differences related to ACE exposure or functional outcomes specifically. A further limitation concerns non-independence between ACE risk and the protective factor “feeling loved” because it was a reverse-scored CTS item contributing to ACE totals, and its inability to capture the broader multidimensional construct of benevolent childhood experiences (e.g., peer relationships, community support, sense of safety) assessed by validated instruments such as the Benevolent Childhood Experiences scale (31); future studies should employ such multi-item measures to more comprehensively assess environmental protective factors. Additionally, we lacked data on medication and PTSD diagnoses despite their relevance to ACE-related long-term outcomes. A further limitation is that polygenic resilience was assessed using a SCZ-resilience PRS. Although transdiagnostic resilience mechanisms are plausible given the shared genetic architecture across psychiatric disorders, it remains unclear whether this score reflects resilience beyond schizophrenia-related liability.

Our findings show that ACEs and feeling loved affect functional outcomes in affective-to-psychotic spectrum disorders, especially occupational stability. They underscore the need to prevent ACEs and foster positive childhood experiences to support socioeconomic stability in people with SMI. Employment support (e.g., individual placement and support) should adopt ACE-informed approaches to help individuals with SMI and ACE histories build and maintain stable workplace relationships. In personal relationships, family interventions are also needed, as illness severity strongly strains these relationships.

## Data Availability

The datasets presented in this article are not readily available because of data protection restrictions concerning the privacy of study participants. Requests to access the datasets should be directed to the corresponding author, subject to a data sharing agreement and approval by the responsible ethics committee.
