# Supplementary material for: Adverse childhood experiences and functional stability in the affective-to-psychotic spectrum: a longitudinal multistate analysis of employment and relationship outcomes
Source: Front Psychiatry. 2026 Oct 6;17:1930635. doi: 10.3389/fpsyt.2026.1930635 (PMC13680451; doi:10.3389/fpsyt.2026.1930635)
Supplement: Supplementary file 1 [file SupplementaryFile1.pdf]

**Supplementary Material 1** Genetic Analyses

Quality control procedures were performed with PLINK v1.9/v2 (1). Single-nucleotide polymorphisms (SNPs) were excluded if they had a call rate below 98% or a minor allele frequency (MAF) below 0.5% or if they deviated significantly from Hardy-Weinberg equilibrium ($p < 0.0001$). On an individual level, participants were excluded if they had a call rate below 98%, were identified as duplicate samples via pairwise identity-by-descent analysis, displayed substantial deviations in heterozygosity, or were determined by multidimensional scaling (MDS) analysis to be of non-European ancestry. The MDS analysis was performed with PLINK v1.9 to assess genetic ancestry and extracted the first ten principal components (PCs). The first two PCs—PC1 and PC2—were incorporated as covariates in subsequent analyses. Furthermore, SNPs with palindromic sequences or a substantial MAF deviation (> 10%) compared with European reference populations were also removed.

Genotype imputation was performed with the Haplotype Reference Consortium panel (2) via the Michigan Imputation Server (3). Post-imputation quality control steps involved the exclusion of SNPs with an imputation quality score ($R^2$) below 0.3 or an MAF below 1%. After imputation, genotype dosage data were used to compute polygenic resilience scores (PRS) for schizophrenia by applying summary statistics from corresponding genome-wide association studies (4). Posterior SNP effect sizes were estimated with polygenic risk score – continuous shrinkage (5) under continuous shrinkage priors. The automatic mode was selected ('*auto*' settings), and the global shrinkage parameter ($\varphi$) was inferred via a fully Bayesian approach.

## References


1. Chang CC, Chow CC, Tellier LC, Vattikuti S, Purcell SM, Lee JJ. Second-generation PLINK: rising to the challenge of larger and richer datasets. *Gigascience* (2015) 4:7. doi: 10.1186/s13742-015-0047-8

2. McCarthy S, Das S, Kretzschmar W, Delaneau O, Wood AR, Teumer A, Kang HM, Fuchsberger C, Danecek P, Sharp K, et al. A reference panel of 64,976 haplotypes for genotype imputation. *Nat Genet* (2016) 48:1279–1283. doi: 10.1038/ng.3643

3. Das S, Forer L, Schönherr S, Sidore C, Locke AE, Kwong A, Vrieze SI, Chew EY, Levy S, McGue M, et al. Next-generation genotype imputation service and methods. *Nat Genet* (2016) 48:1284–1287. doi: 10.1038/ng.3656

4. Hess JL, Mattheisen M, Consortium the SWG of the PG, Greenwood TA, Tsuang MT, Edenberg HJ, Holmans P, Faraone SV, Glatt SJ. A polygenic resilience score moderates the

genetic risk for schizophrenia: Replication in 18,090 cases and 28,114 controls from the Psychiatric Genomics Consortium. *American Journal of Medical Genetics Part B: Neuropsychiatric Genetics* (2024) 195:e32957. doi: 10.1002/ajmg.b.32957

5. Ge T, Chen C-Y, Ni Y, Feng Y-CA, Smoller JW. Polygenic prediction via Bayesian regression and continuous shrinkage priors. *Nat Commun* (2019) 10:1776. doi: 10.1038/s41467-019-09718-5

**Supplementary Material Table 1** *Hazard ratios of losing or finding a job in the group exposed to adverse childhood experiences and in the reference group*
***a)*** *Basic models (corrected for age and sex);* ***b)*** *extended models (corrected for age, sex, Clinical Global Impressions scale, clinical vs. control status, and polygenic resilience score for schizophrenia)*

| | **Basic model** | | | **Extended model** | | |
|---|---|---|---|---|---|---|
| **Transition** | ***Variables*** | ***HR (95% CI)*** | ***p value*** | ***Variables*** | ***HR (95% CI)*** | ***p value*** |
| ***Emotional neglect*** | | | | | | |
| *Job → No Job* | Emotional neglect: Yes vs. No | 1.26 (0.73; 2.16) | 0.4122 | Emotional neglect: Yes vs. No | 1.23 (0.70; 2.14) | 0.4688 |
| | Age per 10 years | 0.79 (0.63; 0.99) | 0.0367* | Age per 10 years | 0.74 (0.58; 0.95) | 0.0164* |
| | Male vs. Female | 1.28 (0.75; 2.17) | 0.3658 | Male vs. Female | 1.08 (0.61; 1.92) | 0.7881 |
| | | | | CGI | 0.92 (0.51; 1.66) | 0.7904 |
| | | | | Clinical vs. control | 4.05 (1.39; 12.09) | 0.0120* |
| No Job → Job | Emotional neglect: Yes vs. No | 1.26 (0.74; 2.12) | 0.3936 | Emotional neglect: Yes vs. No | 1.37 (0.79; 2.37) | 0.2594 |
| | Age per 10 years | 0.65 (0.53; 0.81) | 0.0001* | Age per 10 years | 0.67 (0.53; 0.83) | 0.0004* |
| | Male vs. Female | 1.04 (0.62; 1.74) | 0.8748 | Male vs. Female | 1.10 (0.65; 1.86) | 0.7318 |
| | | | | CGI | 0.54 (0.33; 0.91) | 0.0202* |
| | | | | Clinical vs. control | 1.34 (0.44; 4.05) | 0.6044 |
| **Physical Abuse** | | | | | | |
| Job → No Job | Physical abuse: Yes vs. No | 1.39 (0.80; 2.44) | 0.2451 | Physical abuse: Yes vs. No | 1.32 (0.75; 2.32) | 0.3366 |
| | Age per 10 years | 0.77 (0.61; 0.97) | 0.0253* | Age per 10 years | 0.73 (0.57; 0.94) | 0.0135* |
| | Male vs. Female | 1.33 (0.78; 2.26) | 0.2905 | Male vs. Female | 1.15 (0.65; 2.04) | 0.6293 |
| | | | | CGI | 0.87 (0.48; 1.56) | 0.6390 |
| | | | | Clinical vs. control | 4.40 (1.49; 13.02) | 0.0075* |
| No Job → Job | Physical abuse: Yes vs. No | 1.28 (0.73; 2.22) | 0.3908 | Physical abuse: Yes vs. No | 1.31 (0.74; 2.32) | 0.3469 |
| | Age per 10 years | 0.64 (0.51; 0.81) | 0.0001* | Age per 10 years | 0.66 (0.52; 0.83) | 0.0004* |
| | Male vs. Female | 1.06 (0.64; 1.77) | 0.8224 | Male vs. Female | 1.11 (0.65; 1.87) | 0.7083 |
| | | | | CGI | 0.55 (0.33; 0.91) | 0.0209* |
| | | | | Clinical vs. Control | 1.45 (0.48; 4.34) | 0.5109 |
| **Emotional Abuse** | | | | | | |
| Job → No Job | Emotional abuse: Yes vs. No | 1.44 (0.84; 2.46) | 0.1884 | Emotional abuse: Yes vs. No | 1.48 (0.86; 2.54) | 0.1560 |
| | Age per 10 years | 0.81 (0.64; 1.02) | 0.0687 | Age per 10 years | 0.75 (0.59; 0.97) | 0.0257* |
| | Male vs. Female | 1.24 (0.73; 2.11) | 0.4182 | Male vs. Female | 1.11 (0.63; 1.95) | 0.7287 |
| | | | | CGI | 0.85 (0.47; 1.52) | 0.5772 |
| | | | | Clinical vs. Control | 4.71 (1.58; 14.06) | 0.0055* |
| No Job → Job | Yes vs. No | 0.56 (0.33; 0.95) | 0.0305* | Emotional abuse: Yes vs. No | 0.62 (0.36; 1.06) | 0.0781 |
| | Age per 10 years | 0.64 (0.51; 0.79) | <0.0001* | Age per 10 years | 0.65 (0.52; 0.82) | 0.0002* |
| | Male vs. Female | 1.02 (0.60; 1.71) | 0.9555 | Male vs. Female | 1.08 (0.63; 1.85) | 0.7820 |
| | | | | CGI | 0.54 (0.32; 0.91) | 0.0217* |
| | | | | Clinical vs. Control | 1.57 (0.52; 4.80) | 0.4249 |
| **Sexual Abuse** | | | | | | |
| Job → No Job | Sexual abuse: Yes vs. No | 0.95 (0.52; 1.69) | 0.8280 | Sexual abuse: Yes vs. No | 0.88 (0.49; 1.58) | 0.6747 |
| | Age per 10 years | 0.79 (0.63; 0.99) | 0.0421* | Age per 10 years | 0.75 (0.58; 0.95) | 0.0190* |
| | Male vs. Female | 1.28 (0.73; 2.24) | 0.3854 | Male vs. Female | 1.08 (0.60; 1.94) | 0.7992 |
| | | | | CGI | 0.91 (0.51; 1.62) | 0.7481 |
| | | | | Clinical vs. Control | 4.19 (1.42; 12.36) | 0.0094* |
| No Job → Job | Sexual abuse: Yes vs. No | 0.61 (0.34; 1.08) | 0.0908 | Sexual abuse: Yes vs. No | 0.63 (0.35; 1.13) | 0.1220 |
| | Age per 10 years | 0.67 (0.55; 0.84) | 0.0003* | Age per 10 years | 0.69 (0.55; 0.87) | 0.0013* |
| | Male vs. Female | 0.98 (0.58; 1.65) | 0.9393 | Male vs. Female | 1.03 (0.61; 1.76) | 0.0430* |
| | | | | CGI | 0.58 (0.35; 0.98) | 0.6826 |
| | | | | Clinical vs. Control | 1.26 (0.41; 3.85) | 0.7562 |
| **Physical Neglect** | | | | | | |
| Job → No Job | Physical neglect: Yes vs. No | 1.37 (0.75; 2.49) | 0.3014 | Physical neglect: Yes vs. No | 1.39 (0.76; 2.56) | 0.2858 |
| | Age per 10 years | 0.77 (0.61; 0.97) | 0.0293* | Age per 10 years | 0.72 (0.56; 0.93) | 0.0114* |
| | Male vs. Female | 1.23 (0.72; 2.10) | 0.4569 | Male vs. Female | 1.09 (0.62; 1.93) | 0.7608 |
| | | | | CGI | 0.84 (0.47; 1.50) | 0.5561 |
| | | | | Clinical vs. Control | 4.63 (1.56; 13.70) | 0.0057* |
| No Job → Job | Yes vs. No | 0.89 (0.47; 1.69) | 0.7155 | Physical neglect: Yes vs. No | 0.93 (0.48; 1.79) | 0.8239 |
| | Age per 10 years | 0.67 (0.54; 0.83) | 0.0002* | Age per 10 years | 0.68 (0.54; 0.85) | 0.0008* |
| | Male vs. Female | 1.03 (0.61; 1.73) | 0.9179 | Male vs. Female | 1.09 (0.64; 1.85) | 0.7640 |
| | | | | CGI | 0.54 (0.32; 0.91) | 0.0212* |
| | | | | Clinical vs. Control | 1.47 (0.48; 4.51) | 0.4986 |

CGI, Clinical Global Impressions scale (CGI scores were grouped into three categories: (1) 1–2 = healthy/mildly ill; (2) 3–4 = moderately ill; (3) 5–7 = severely ill); CI, confidence interval; HR, hazard ratio
* Significant result ($p < 0.05$)

**Supplementary Material Table 2** *Hazard ratios of losing or finding a partner in the group exposed to adverse childhood experiences and in the reference group*
***a)*** *Basic model (corrected for age and sex);* ***b)*** *extended model (corrected for age, sex, Clinical Global Impressions scale, clinical vs. control status, and polygenic resilience score for schizophrenia)*

| | Basic model | | | Extended model | | |
|---|---|---|---|---|---|---|
| **Transition** | ***Variables*** | ***HR (95% CI)*** | ***p value*** | ***Variables*** | ***HR (95% CI)*** | ***p value*** |
| **Emotional neglect** | | | | | | |
| Partner → No Partner | Emotional neglect: Yes vs. No | 1.67 (0.91; 3.08) | 0.0999 | Emotional neglect: Yes vs. No | 1.51 (0.81; 2.80) | 0.1920 |
| | Age per 10 years | 0.64 (0.49; 0.82) | 0.0006* | Age per 10 years | 0.60 (0.45; 0.80) | 0.0006* |
| | Male vs. Female | 0.92 (0.50; 1.70) | 0.7887 | Male vs. Female | 0.77 (0.41; 1.47) | 0.4332 |
| | | | | CGI | 1.86 (1.00; 3.48) | 0.0512 |
| | | | | Clinical vs. control | 0.88 (0.25; 3.05) | 0.8395 |
| No Partner → Partner | Emotional neglect: Yes vs. No | 1.46 (0.75; 2.83) | 0.2661 | Emotional neglect: Yes vs. No | 1.43 (0.73; 2.78) | 0.2953 |
| | Age per 10 years | 0.63 (0.48; 0.83) | 0.0012* | Age per 10 years | 0.62 (0.47; 0.83) | 0.0010* |
| | Male vs. Female | 0.57 (0.29; 1.10) | 0.0946 | Male vs. Female | 0.54 (0.28; 1.06) | 0.0719 |
| | | | | CGI | 1.26 (0.66; 2.40) | 0.4754 |
| | | | | Clinical vs. control | 1.00 (0.25; 3.96) | 0.9984 |
| **Physical Abuse** | | | | | | |
| Partner → No Partner | Physical abuse: Yes vs. No | 1.50 (0.77; 2.92) | 0.2374 | Physical abuse: Yes vs. No | 1.12 (0.56; 2.25) | 0.7437 |
| | Age per 10 years | 0.62 (0.47; 0.81) | 0.0005* | Age per 10 years | 0.59 (0.44; 0.80) | 0.0006* |
| | Male vs. Female | 0.98 (0.53; 1.81) | 0.9525 | Male vs. Female | 0.82 (0.43; 1.54) | 0.5290 |
| | | | | CGI | 1.81 (0.95; 3.46) | 0.0700 |
| | | | | Clinical vs. control | 0.97 (0.28; 3.34) | 0.9610 |
| No Partner → Partner | Physical abuse: Yes vs. No | 0.91 (0.47; 1.76) | 0.7799 | Physical abuse: Yes vs. No | 0.80 (0.40; 1.61) | 0.5343 |
| | Age per 10 years | 0.63 (0.47; 0.85) | 0.0025* | Age per 10 years | 0.66 (0.50; 0.87) | 0.0034* |
| | Male vs. Female | 0.64 (0.32; 1.28) | 0.2081 | Male vs. Female | 0.50 (0.26; 0.97) | 0.0417* |
| | | | | CGI | 1.19 (0.64; 2.23) | 0.5815 |
| | | | | Clinical vs. Control | 1.14 (0.30; 4.36) | 0.8517 |
| **Emotional Abuse** | | | | | | |
| Partner → No Partner | Emotional abuse: Yes vs. No | 2.33 (1.23; 4.41) | 0.0096* | Emotional abuse: Yes vs. No | 2.06 (1.08; 3.94) | 0.0285* |
| | Age per 10 years | 0.68 (0.52; 0.88) | 0.0030* | Age per 10 years | 0.63 (0.47; 0.84) | 0.0021* |
| | Male vs. Female | 0.99 (0.53; 1.84) | 0.9781 | Male vs. Female | 0.85 (0.45; 1.61) | 0.6621 |
| | | | | CGI | 1.84 (0.96; 3.51) | 0.0647 |
| | | | | Clinical vs. Control | 0.88 (0.25; 3.13) | 0.8439 |
| No Partner → Partner | Yes vs. No | 1.45 (0.66; 3.20) | 0.3624 | Emotional abuse: Yes vs. No | 1.85 (0.91; 3.76) | 0.0869 |
| | Age per 10 years | 0.62 (0.46; 0.85) | 0.0027* | Age per 10 years | 0.67 (0.51; 0.88) | 0.0039* |
| | Male vs. Female | 0.55 (0.27; 1.12) | 0.1018 | Male vs. Female | 0.51 (0.26; 1.00) | 0.0497* |
| | | | | CGI | 1.13 (0.61; 2.10) | 0.6970 |
| | | | | Clinical vs. Control | 1.28 (0.34; 4.85) | 0.7147 |
| **Sexual Abuse** | | | | | | |
| Partner → No Partner | Sexual abuse: Yes vs. No | 0.55 (0.27; 1.12) | 0.0980 | Sexual abuse: Yes vs. No | 0.54 (0.27; 1.09) | 0.0861 |
| | Age per 10 years | 0.65 (0.51; 0.84) | 0.0008* | Age per 10 years | 0.60 (0.45; 0.81) | 0.0006* |
| | Male vs. Female | 0.82 (0.44; 1.56) | 0.5516 | Male vs. Female | 0.67 (0.35; 1.30) | 0.2366 |
| | | | | CGI | 1.96 (1.05; 3.68) | 0.0353* |
| | | | | Clinical vs. Control | 0.87 (0.25; 3.02) | 0.8204 |
| No Partner → Partner | Sexual abuse: Yes vs. No | 1.48 (0.78; 2.81) | 0.2298 | Sexual abuse: Yes vs. No | 1.42 (0.73; 2.74) | 0.2970 |
| | Age per 10 years | 0.65 (0.50; 0.85) | 0.0014* | Age per 10 years | 0.64 (0.49; 0.84) | 0.0013* |
| | Male vs. Female | 0.54 (0.28; 1.04) | 0.0645 | Male vs. Female | 0.51 (0.26; 0.99) | 0.0474* |
| | | | | CGI | 1.12 (0.60; 2.08) | 0.7308 |
| | | | | Clinical vs. Control | 1.23 (0.33; 4.66) | 0.7562 |
| **Physical Neglect** | | | | | | |
| Partner → No Partner | Physical neglect: Yes vs. No | 0.54 (0.22; 1.29) | 0.1638 | Physical neglect: Yes vs. No | 0.49 (0.21; 1.19) | 0.1159 |
| | Age per 10 years | 0.62 (0.52; 0.87) | 0.0024* | Age per 10 years | 0.62 (0.46; 0.83) | 0.0014* |
| | Male vs. Female | 0.99 (0.54; 1.83) | 0.9870 | Male vs. Female | 0.81 (0.43; 1.52) | 0.5109 |
| | | | | CGI | 1.93 (1.04; 3.59) | 0.0366* |
| | | | | Clinical vs. Control | 0.94 (0.28; 3.20) | 0.9209 |
| No Partner → Partner | Yes vs. No | 0.80 (0.36; 1.74) | 0.5677 | Physical neglect: Yes vs. No | 0.75 (0.34; 1.65) | 0.4719 |
| | Age per 10 years | 0.67 (0.52; 0.87) | 0.0028* | Age per 10 years | 0.66 (0.50; 0.86) | 0.0022* |
| | Male vs. Female | 0.52 (0.27; 1.00) | 0.0483* | Male vs. Female | 0.49 (0.25; 0.95) | 0.0345* |
| | | | | CGI | 1.18 (0.64; 2.20) | 0.5937 |
| | | | | Clinical vs. Control | 1.24 (0.33; 4.67) | 0.7466 |

CGI, Clinical Global Impressions scale (CGI scores were grouped into three categories: (1) 1–2 = healthy/mildly ill; (2) 3–4 = moderately ill; (3) 5–7 = severely ill); CI, confidence interval; HR, hazard ratio
* Significant result ($p < 0.05$)

**Supplementary Material Figure 1** *Hazard ratios of losing or finding a job in the group exposed to adverse childhood experiences and in the reference group*
***a)*** *Basic models (corrected for age and sex);* ***b)*** *extended models (corrected for age, sex, Clinical Global Impressions scale, clinical vs. control status, and educational status)*

***a)***

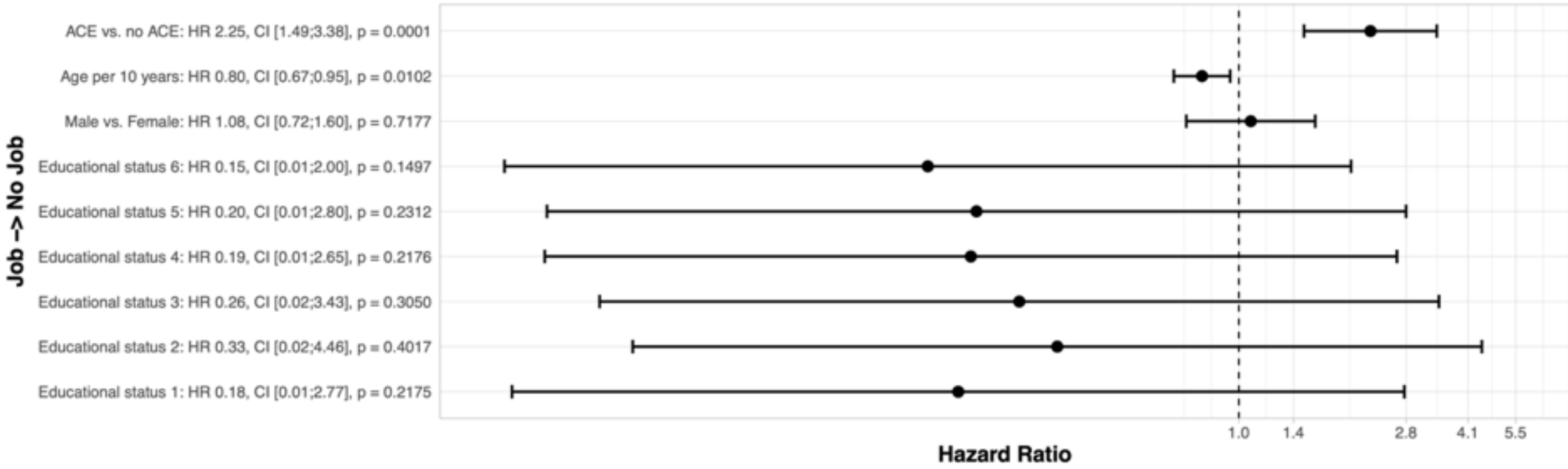


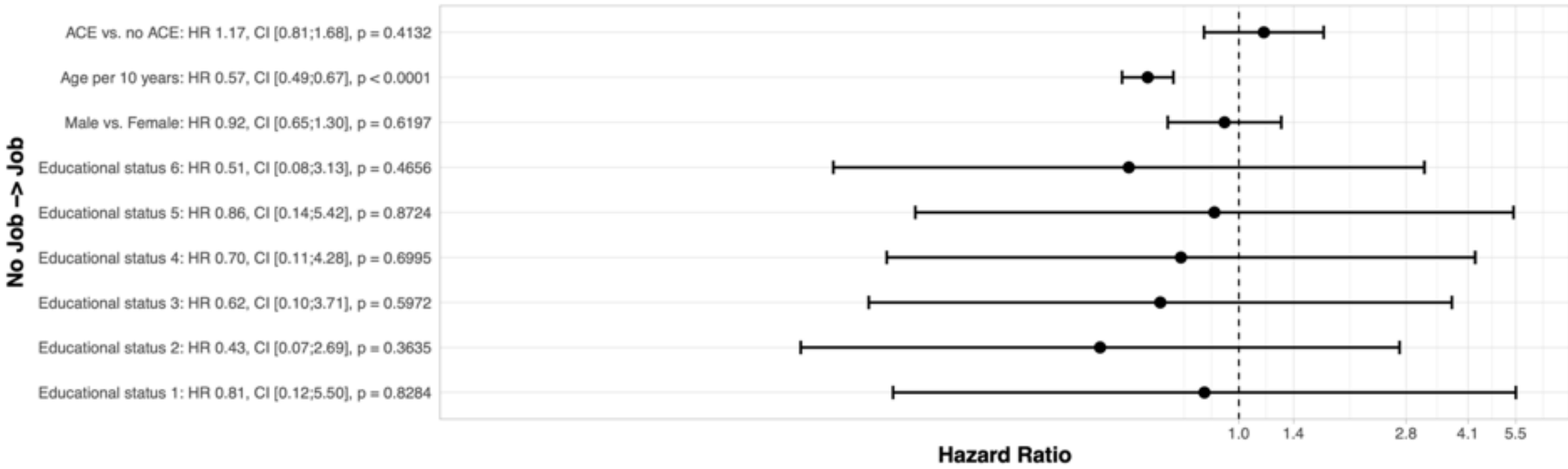


**b)**

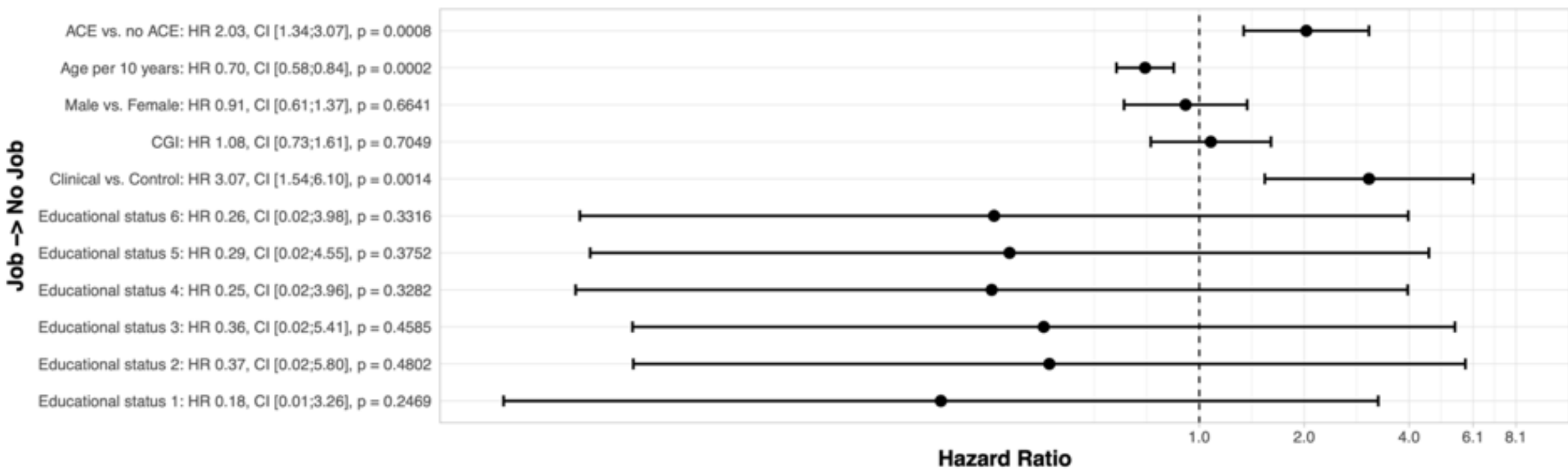


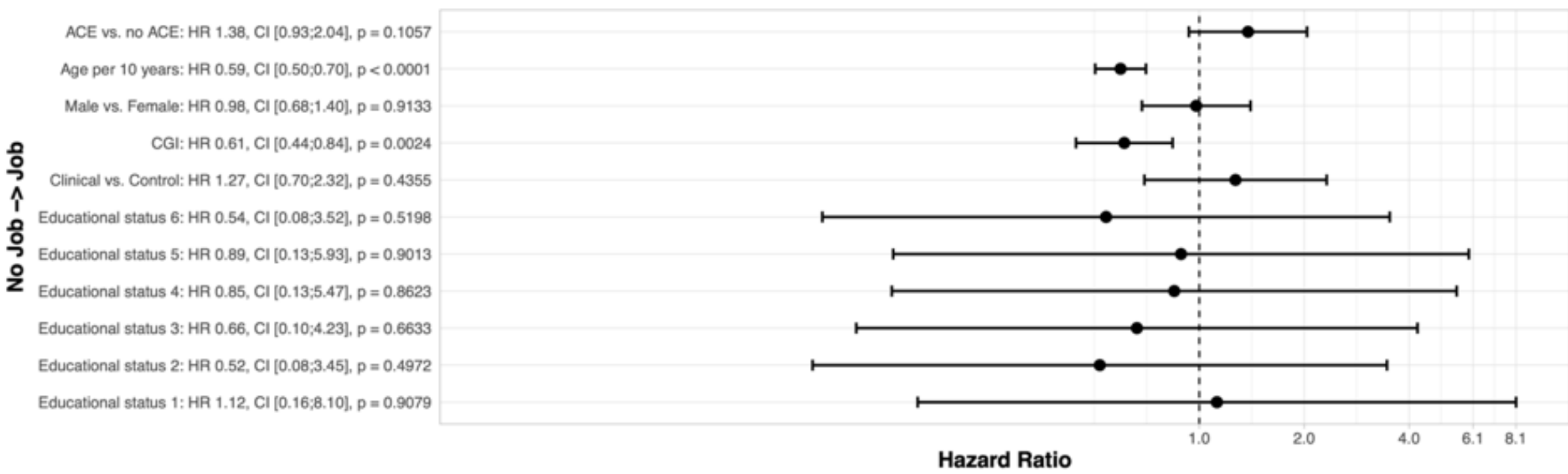


Significance level: p-value < 0.05
Educational status scale: 0, lowest schooling (reference group) or professional education; 6, highest possible academic degree
ACE, adverse childhood experiences; CGI, Clinical Global Impression scale (CGI scores were grouped into three categories: (1) 1–2 = healthy/mildly ill; (2) 3–4 = moderately ill; (3) 5–7 = severely ill); CI, confidence interval; HR, hazard ratio

**Supplementary Material Figure 2** *Hazard ratios of losing or finding a job in the group exposed to adverse childhood experiences and in the reference group*
***a)*** *Basic models (corrected for age and sex);* ***b)*** *extended models (corrected for age, sex, Clinical Global Impressions scale, clinical vs. control status, and educational status)*

**a)**

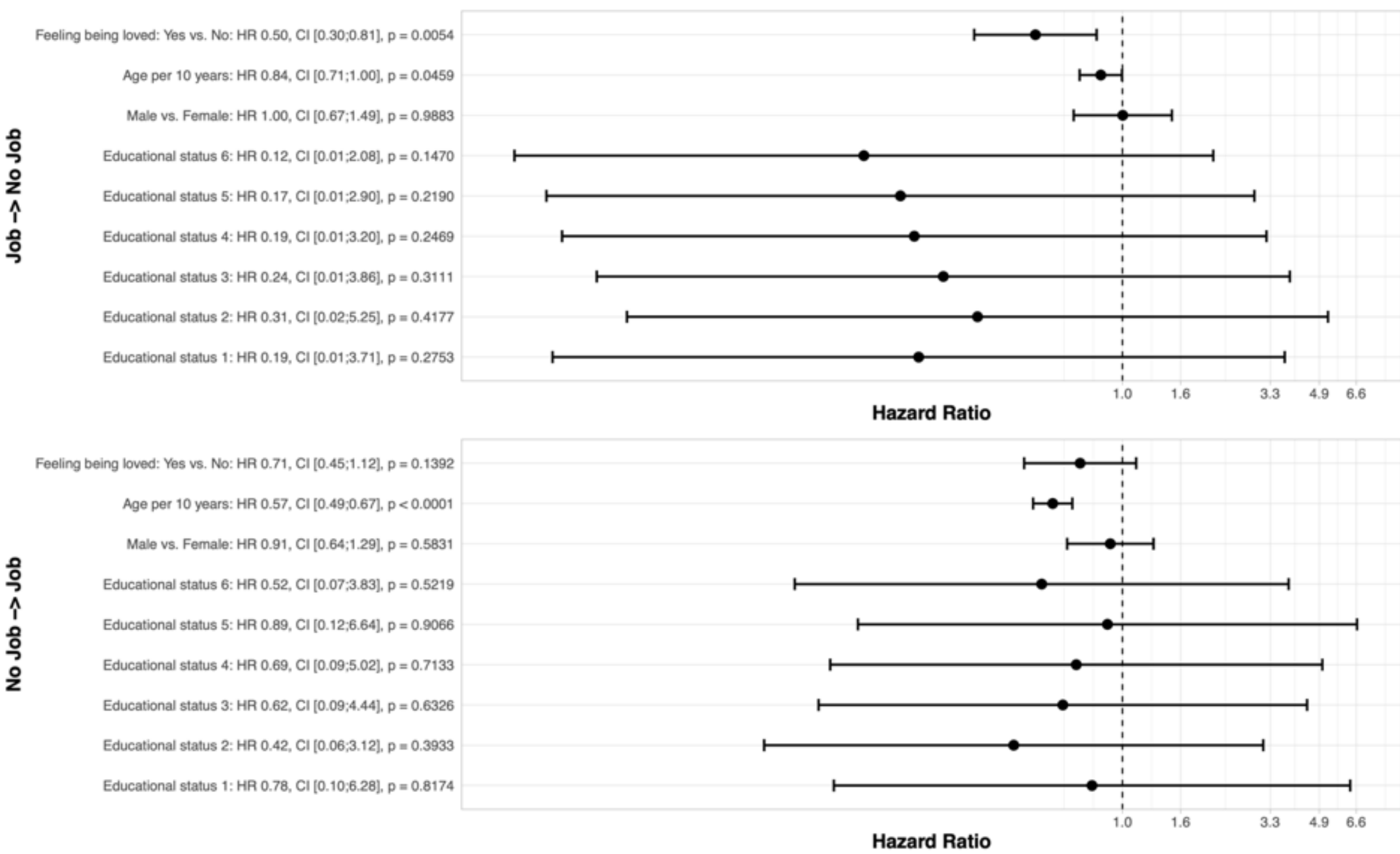


**b)**

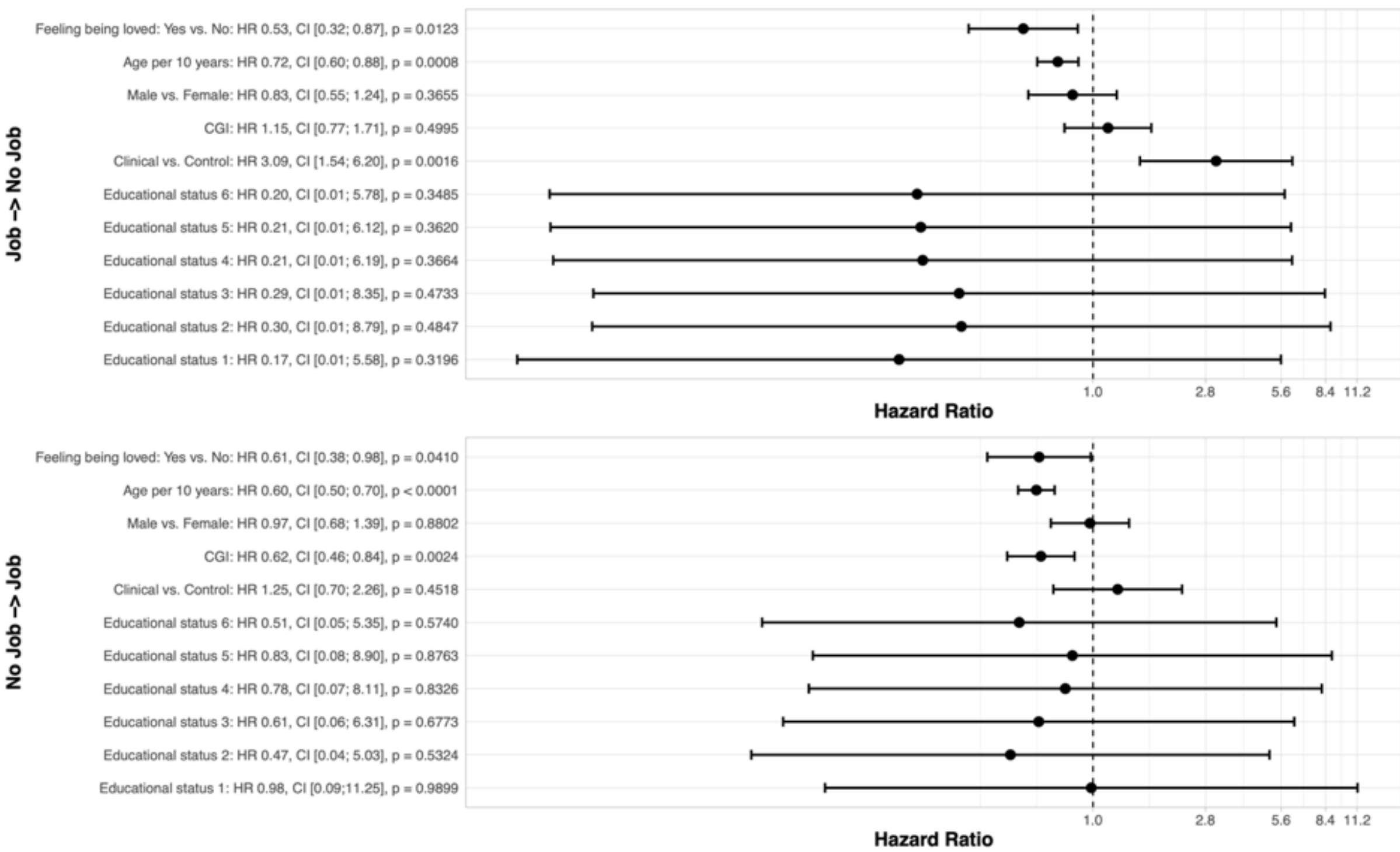


Significance level: p-value < 0.05
Educational status scale: 0, lowest schooling (reference group) no schooling or professional education; 6, highest possible academic degree; CGI, Clinical Global Impressions scale (CGI scores were grouped into three categories: (1) 1–2 = healthy/mildly ill; (2) 3–4 = moderately ill; (3) 5–7 = severely ill); CI, confidence interval; HR, hazard ratio

**Supplementary Material Figure 3** *Hazard ratios of losing or finding a job in the group exposed to adverse childhood experiences and in the reference group*
***a)*** *Basic models (corrected for age and sex);* ***b)*** *extended models (corrected for age, sex, Clinical Global Impressions scale, clinical vs. control status, and polygenic resilience score for schizophrenia)*

**a)**

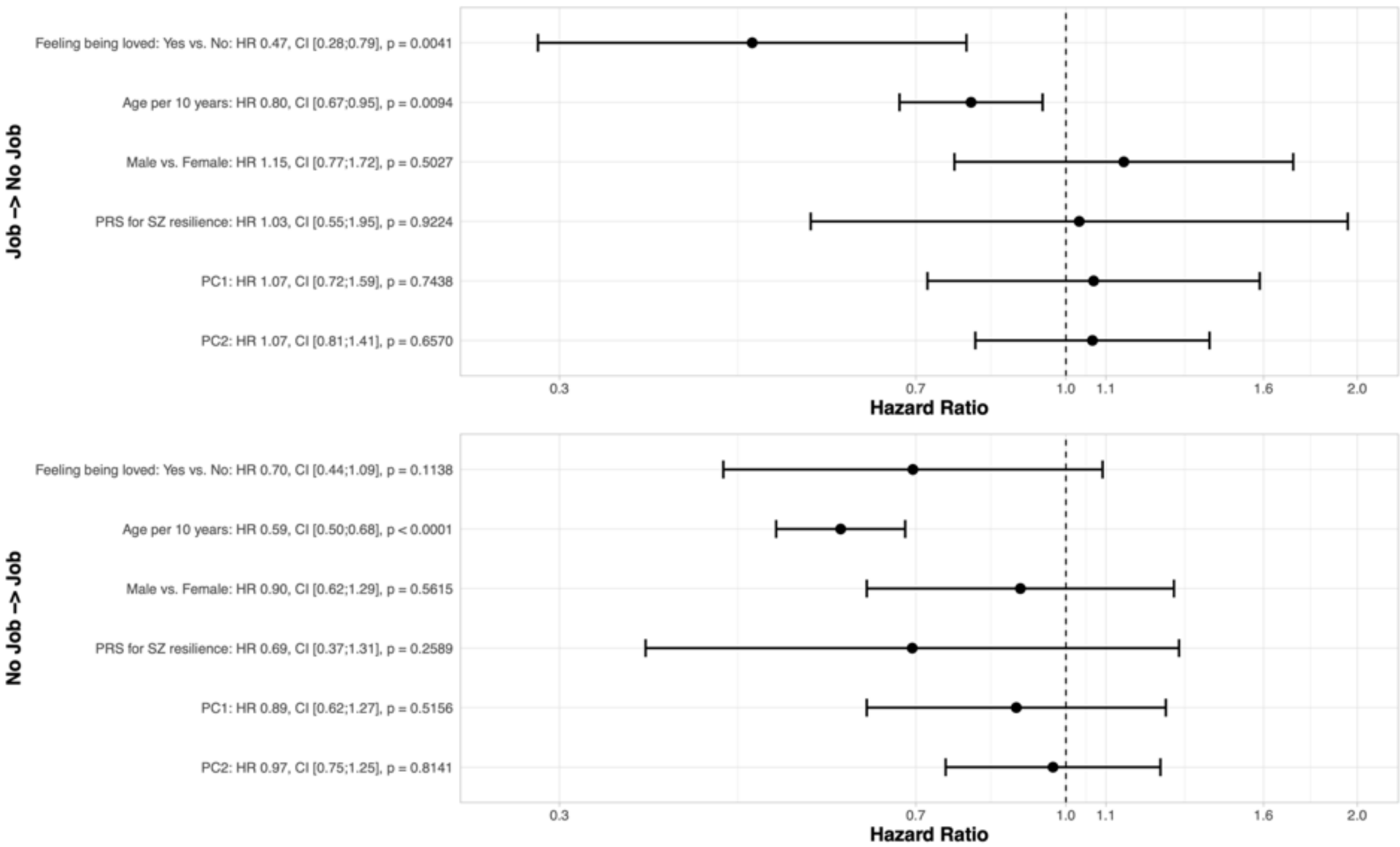


**b)**

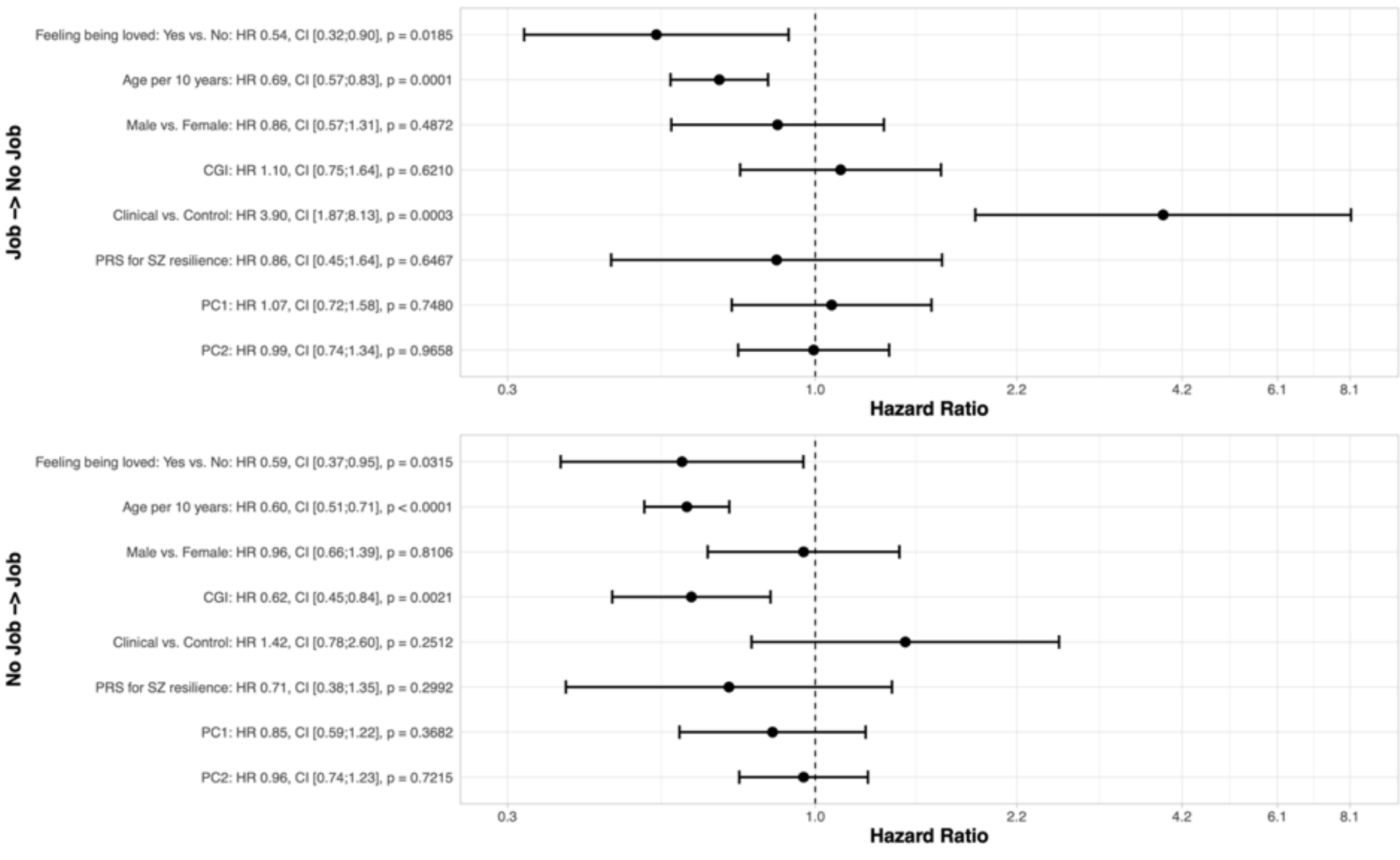


Significance level: p-value < 0.05
CGI, Clinical Global Impressions scale (CGI scores were grouped into three categories: (1) 1–2 = healthy/mildly ill; (2) 3–4 = moderately ill; (3) 5–7 = severely ill); CI, confidence interval; HR, hazard ratio; PC1, Principle component 1; PC2, Principle component 2; PRS, polygenic resilience score; SZ, Schizophrenia

**Supplementary Material Figure 4** *Hazard ratios of losing or finding a partner in the group exposed to adverse childhood experiences and in the reference group*
***a)*** *Basic models (corrected for age and sex);* ***b)*** *extended models (corrected for age, sex, Clinical Global Impressions scale, clinical vs. control status, and polygenic resilience score for schizophrenia)*

**a)**

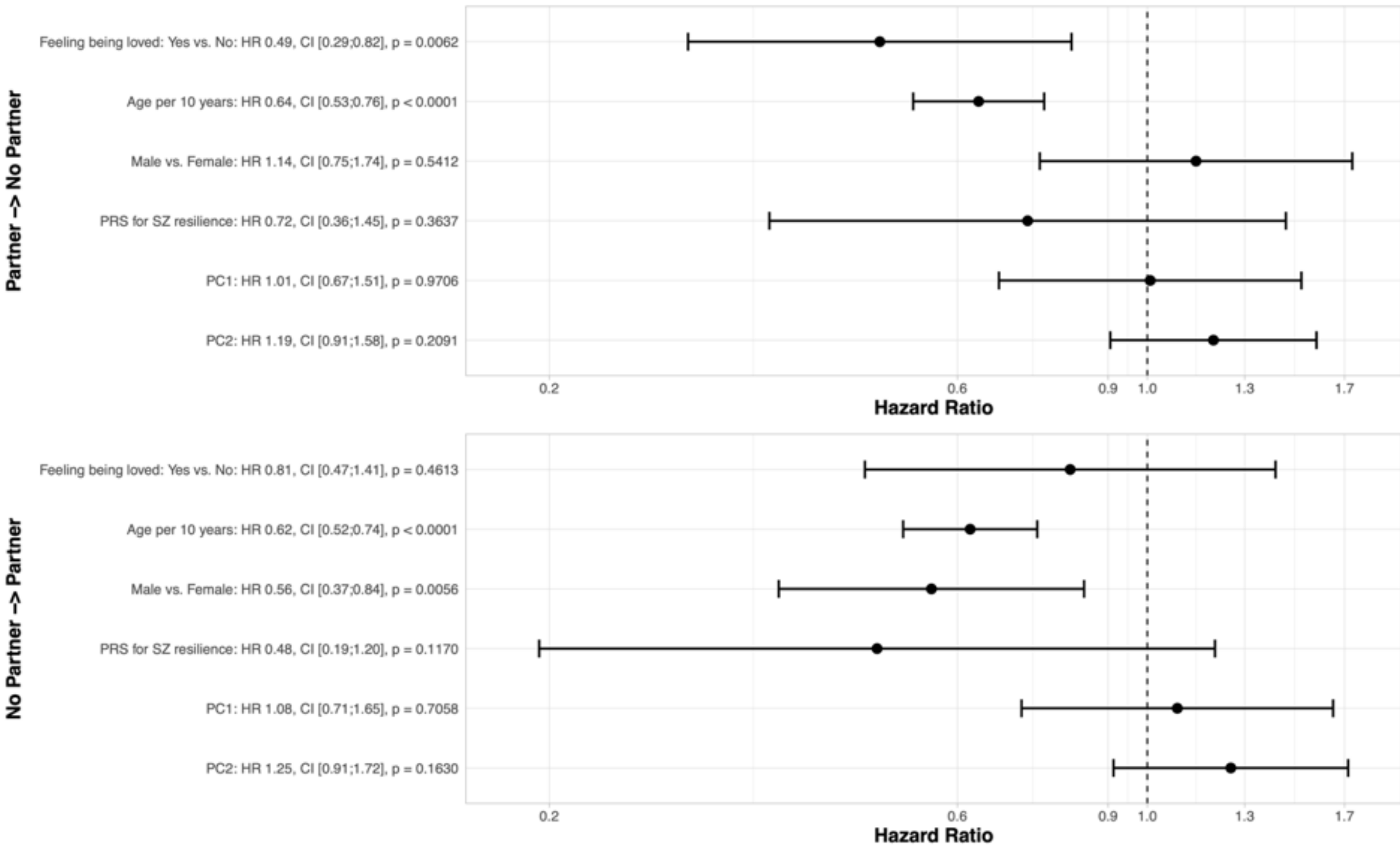


**b)**

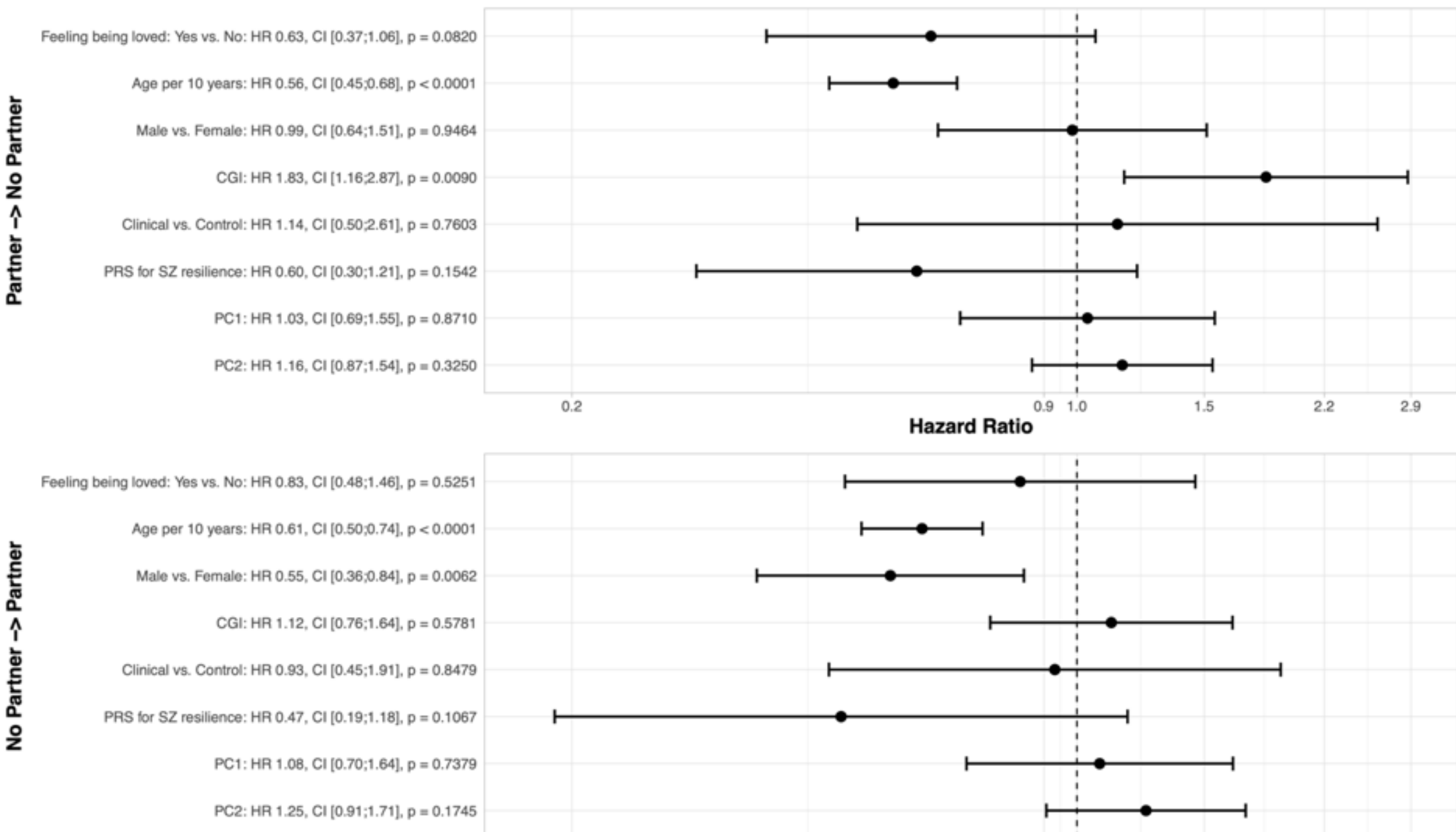


Significance level: p-value < 0.05
CGI, Clinical Global Impressions scale (CGI scores were grouped into three categories: (1) 1–2 = healthy/mildly ill; (2) 3–4 = moderately ill; (3) 5–7 = severely ill); CI, confidence interval; HR, hazard ratio; PC1, Principle component 1; PC2, Principle component 2; PRS, polygenic resilience score; SZ, schizophrenia

**Supplementary Material Figure 5** Hazard ratios in the basic multistate models (corrected for age and sex)

of losing or finding
**a)** a job or
**b)** partner for the subtypes of adverse childhood experiences

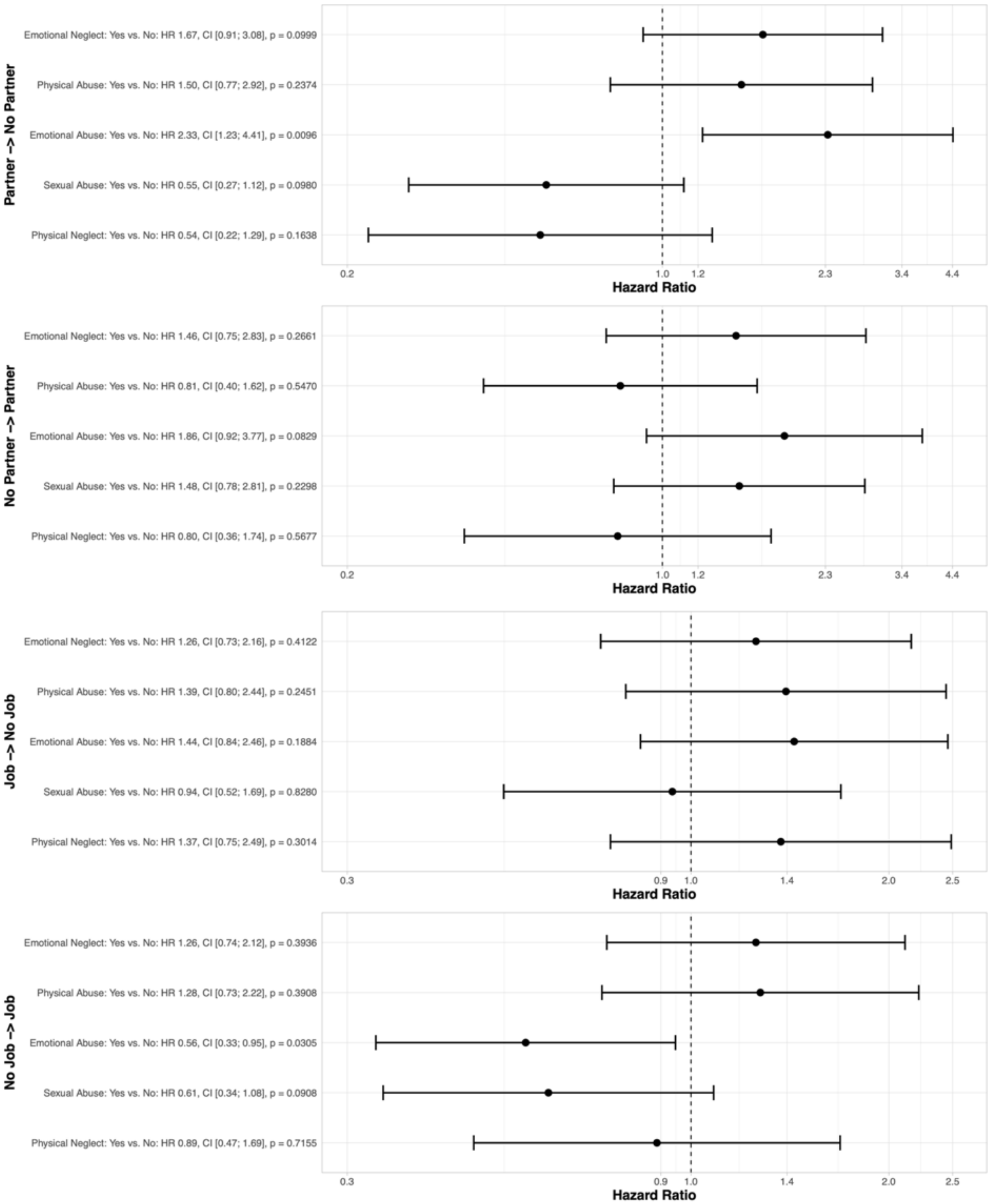


Significance level: p-value < 0.05
CI, confidence interval; HR, hazard ratio
